# Humanized anti-DEspR IgG4^S228P^ antibody increases overall survival in a pancreatic cancer stem cell-xenograft peritoneal carcinomatosis rat^*nu/nu*^ model

**DOI:** 10.1186/s12885-021-08107-w

**Published:** 2021-04-14

**Authors:** Christopher M. Gromisch, Glaiza L. A. Tan, Khristine Amber Pasion, Ann-Marie Moran, Matthew S. Gromisch, Mark W. Grinstaff, Francis J. Carr, Victoria L. M. Herrera, Nelson Ruiz-Opazo

**Affiliations:** 1 Department of Pharmacology, Boston University School of Medicine, Boston, MA USA; 2 Whitaker Cardiovascular Institute and Department of Medicine, Boston University School of Medicine, Boston, MA USA; 3 Abtelum Biomedical, Inc., now NControl Therapeutics, Inc., Boston, MA, USA; 4 Department of Chemistry, Boston University, Boston, MA USA

**Keywords:** Dual endothelin-1/signal peptide receptor, DEspR, Cancer stem cells, Pancreatic cancer, Peritoneal carcinomatosis, Nude rat xenograft tumor model, IgG4 antibody therapy

## Abstract

**Background:**

Pancreatic peritoneal carcinomatosis (PPC), with the worst median overall-survival (mOS), epitomizes the incurability of metastatic cancer. Cancer stem cells (CSCs) underpin this incurability. However, inhibitors of CSC-stemness fail to increase mOS in cancer patients despite preclinical tumor-reduction. This shortfall reinforces that preclinical efficacy should be defined by increased mOS in the presence of cancer comorbidities, CSC-heterogeneity and plasticity. The primary objectives of this study are: to test the dual endothelin-1/signal peptide receptor, DEspR, as a nodal therapeutic target in PPC, given DEspR induction in anoikis-resistant pancreatic CSCs, and to validate humanized anti-DEspR antibody, hu-6g8, as a potential therapeutic for PPC.

**Methods:**

We used heterogeneous pools of CSCs selected for anoikis resistance from reprogrammed Panc1 and MiaPaCa2 tumor cells (TCs), and adherent TCs reprogrammed from CSCs (cscTCs). We used multiple anti-DEspR blocking antibodies (mAbs) with different epitopes, and a humanized anti-DEspR recombinant mAb cross-reactive in rodents and humans, to test DEspR inhibition effects. We measured DEspR-inhibition efficacy on multiple prometastatic CSC-functions in vitro, and on tumorigenesis and overall survival in a CSC-derived xenograft (CDX) nude rat model of PPC with comorbidities.

**Results:**

Here we show that DEspR, a stress-survival receptor, is present on subsets of PDAC Panc1-TCs, TC-derived CSCs, and CSC-differentiated TCs (cscTCs), and that DESpR-inhibition decreases apoptosis-resistance and pro-metastatic mesenchymal functions of CSCs and cscTCs in vitro. We resolve the DNA-sequence/protein-function discordance by confirming ADAR1-RNA editing-dependent DEspR-protein expression in Panc1 and MiaPaCa2 TCs. To advance DEspR-inhibition as a nodal therapeutic approach for PPC, we developed and show improved functionality of a recombinant, humanized anti-DEspR IgG4^S228P^ antibody, hu-6g8, over murine precursor anti-DEspR mabs. Hu-6g8 internalizes and translocates to the nucleus colocalized with cyto-nuclear shuttling galectins-1/3, and induces apoptotic cell changes. DEspR-inhibition blocks transperitoneal dissemination and progression to peritoneal carcinomatosis of heterogeneous DEspR±/CD133 ± Panc1-derived CSCs in xenografted nude rats, improving mOS without chemotherapy-like adverse effects. Lastly, we show DEspR expression in Stage II-IV primary and invasive TCs in the stroma in PDAC-patient tumor arrays.

**Conclusion:**

Collectively, the data support humanized anti-DEspR hu-6g8 as a potential targeted antibody-therapeutic with promising efficacy, safety and prevalence profiles for PPC patients.

**Supplementary Information:**

The online version contains supplementary material available at 10.1186/s12885-021-08107-w.

## Background

Cancer metastasis causes 90% of all cancer-related deaths and remains a high unmet need despite decades of research [1]. Pancreatic ductal adenocarcinoma (PDAC) epitomizes the unmet need with a 44:55 death-to-case ratio per year [2]. While second to PDAC liver metastasis in prevalence, peritoneal metastasis exhibits the worst median overall-survival (mOS) of all PDAC metastases [3] with rapid feed-forward dissemination-progression to pancreatic peritoneal carcinomatosis (PPC) [4]. Development of PPC after curative-intent surgery, despite post-resection adjuvant therapy, especially in patients with tumor cell-positive peritoneal fluid cytology 6g[5], indicates inherent PPC therapy-resistance that is not simply due to delayed diagnosis. PPC does not benefit from surgical debulking, unlike colorectal/ovarian peritoneal metastases [6], thus reiterating the therapeutic challenges and high unmet need in PPC.

Cumulative research implicates cancer stem cells (CSCs) [7] in post-resection cancer recurrence and metastasis [8, 9], especially in PDAC [8]. However, the failure to meet primary endpoints in clinical trials of “CSC-only” inhibitors indicates the need for inhibition of both CSCs and TCs [9] in order to address therapy-resistance arising from bi-directional CSC/TC-reprogramming or plasticity, and CSC/TC heterogeneity [9, 10]. The dual endothelin-1/signal peptide^VEGF^ receptor (DEspR) is a cell-surface accessible target induced on subsets of multi-potential, highly tumorigenic PDAC (Panc1-derived) CSCs and TCs [11]. DEspR-inhibition decreases CSC anoikis resistance, stress-survival, vasculo-angiogenesis, and RNA levels of pro-survival Mcl1 and cIAP2 proteins [11]. PDAC TCs express both DEspR ligands, endothelin-1 (ET1) [12] and signal peptide of VEGF (SP^VEGF^) cleaved from the VEGF-propeptide [13]. Expression levels of both ET1 and VEGF (and by extension SP^VEGF^) are associated with aggressive PDAC and poor outcomes [12, 13]. However, while both ET1 and VEGF-axes are implicated in PDAC progression, inhibitors of ET1-A and/or ET1-B receptor [14] or the VEGF/receptor-axis [15] are not FDA-approved targeted therapies for PDAC. By hypotheses elimination, DEspR, activated by ET1 and SP^VEGF^, remains the missing receptor pathway to inhibit when ET1 and VEGF and perforce, SP^VEGF^, are elevated.

DEspR is indeed a ‘missing puzzle piece’ as it is annotated as a non-coding gene, due to a stop codon [T-G-A] in the NCBI DNA database [16], in lieu of tryptophan-codon [T-G-G] at amino acid position-#14 (tryp#14). Given multiple experimental evidence detecting DEspR protein, functionality, and RNA-sequences with [T-G-A/G]-tryp#14 in placenta RNA-seq dataset [11, 17, 18], analysis for potential ADAR1 RNA-editing would clarify this DNA-protein discordance.

Here we show that DEspR, a stress-survival receptor, induced on CSCs and expressed in CSC-derived tumor cells, is a nodal therapeutic target in PPC. To advance a potential therapy, we developed, tested and validate the humanized anti-DEspR IgG4^S228P^ antibody as a potential targeted therapy for patients with PPC with a promising preclinical efficacy and safety profile, and a clinically relevant expression profile in primary and metastatic pancreatic cancer.

## Materials and methods

Please see Supplementary Methods and Materials (Additional file 1) for specifics.

### Study design

The purpose of this study was to investigate anti-DEspR therapy as a translatable pathway for curative-intent therapy for PPC. DEspR-inhibition was attained using different anti-DEspR antibodies binding to their respective epitopes on human-DEspR. In vitro experiments were designed to assess the impact of DEspR-inhibition on TC and CSC pro-tumor stress survival and functionalities. Two PDAC cell lines were used representing different KRAS mutations: Panc1 with G12D mutant KRAS detected in 70–95% of PDAC cases, and MiaPaCa2 PaCa2 with G12C mtKRAS detected in 1–3% of PDAC cases. Tumor cells and CSCs comprised different subsets of permutations of DEspR±/CD133 ± in order to represent heterogeneity. Studies were done on all key components of the CSC-TC spectrum: DEspR± CSCs, TCs, and cscTCs, in order to demonstrate efficacy of DEspR-inhibition regardless of CSC/TC plasticity. Independent biological replicates were performed on different days with different experimenters, and technical replicates were performed in triplicate to demonstrate methodological rigor, unless otherwise stated. Different informative, functional endpoints were selected to affirm reproducibility of DEspR-inhibition efficacy in vitro.

In vivo experiments in CSC-derived xenograft (CDX)-subcutaneous and PPC nude rat models evaluated the impact of DEspR-inhibition on tumorigenicity and progression, and overall survival in female and male PPC rats. PK/PD experiments were performed to better characterize anti-DEspR therapeutics. Outbred Rowett nude rat models can attain larger allowable tumors (20% of 250 g BW), hence longer timecourse with more complex tumors and cancer comorbidities, compared to tumor-to-bodyweight ratio limits in inbred nude mice (20% of 25 g BW). Contemporary age-matched controls were used. Treatment-group assignments were based on pre-study defined distribution scheme that ascertained that rats in treated and control groups were litter-matched to the best possible, of identical ages, equivalent weights, and received treatment/mock-treatment injections under identical conditions. All animals were monitored by blinded Lab Animal Science (LASC) technicians, with prior IACUC approved study-endpoints without modification. Sample size was calculated based on pilot studies in order to allow significant statistical power to assess primary endpoints. DEspR expression in human PDAC tumor arrays was quantified in blinded manner. Tumor cores, in duplicates, represented different PDAC stages of disease. All available data points were included in analysis.

### Cancer cell lines and cancer stem-like cells (CSCs)

We used three human pancreatic cancer cell lines: Panc1 (CRL-1469), MiaPaCa2 (CRL-1420), Capan-1 (HTB-79) cells, and one normal endothelial cell line, HUVEC (CRL-1730), which were obtained from the American Type Culture Collection (ATCC, Manassas, VA) and grown according to ATCC recommendations. All CSCs were reprogrammed from TCs by selective growth in ultra-low adherent 100 mm plates (Corning, 3261), comprised of mixed molecular-type subsets (DEspR±, CD133±), cryogenically preserved after 3 passages, and proven increased tumorigenesis after 5 passages, as previously described [11]. Panc1-CSCs were differentiated to TCs by plating Panc1 CSCs on Lab-Tek II Chamber Slides (Nunc, 154526PK) in TC cell culture media per ATCC protocols for Panc1 and MiaPaCa2 respectively.

### Anti-DEspR antibody design

**Fig. 1:**
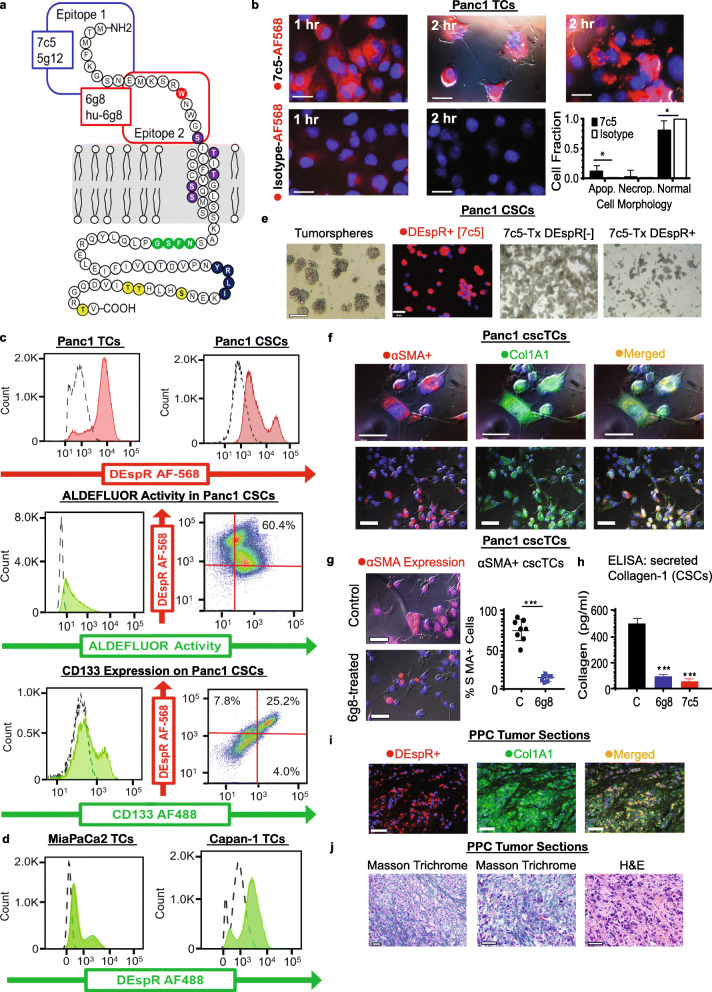
DEspR modulates survival and functionality of Panc1-CSCs, cscTCs, and TCs. **a** DEspR-protein diagram showing epitope-1 and epitope-2 with corresponding anti-DEspR mAbs 7c5, 5g12, 6g8, hu-6g8; consensus sequences for glycosylation (green), internalization recognition sequence (navy), O-glycosylation (purple), and phosphorylation (yellow). **b** Representative images showing internalization of fluorescent AF568-labeled 7c5 and AF568-IgG4 isotype-control in Panc1-TCs after 1- and 2-h, and associated apoptotic (Apop.) and necroptotic (Necrop.) cell morphology changes. Chi-square test for independence, two-tailed t-test p<0.0001; 7c5-treated n=280 cells, isotype-treated n=242 cells. **c** Flow-cytometry showing **[Top]:** DEspR cell-surface expression on Panc1-TCs (65–75%) and Panc1-CSCs (60–80%) [dashed-line isotype vs. red AF-568-7c5]; **[Middle]:** ALDEFLUOR activity/ expression in Panc1-CSCs (68–72%) [dashed-line DEAB control vs green ALDEFLUOR] and ALDEFLUOR expression of DEspR+ Panc1-CSCs (58.2–60.4%); **[Bottom]**: CD133 expression on Panc1 CSCs (28–34%) [dashed-line isotype vs. green AF-488-CD133] and DEspR+/CD133+ CSCs (24.1%). **d** Flow-cytometry showing DEspR cell-surface expression on MiaPaca2-TCs (58–65%) and Capan-1-TCs (31–40%) [dashed-line isotype vs. green AF-488-7c5]. **e** Panc1-CSC tumorspheres (Bar: 100 μm) with staining of DEspR+ Panc1-CSCs from dispersed tumorspheroids. After 30-min 7c5-AF568 binding, then MoFlo-sorting and plating in low-adherence cultures, phase contrast image shows viable DEspR[−] CSCs **(7c5-Tx DEspR[−])** but no spheroid-formation; in contrast to minimal to non-thriving 7c5-sorted/inhibited DEspR+ CSCs **(7c5-Tx DEspR+)**. **f** Panc1-cscTCs co-express αSMA (red) and Col1A1(green), merged (yellow) (top bar: 20 μm, bottom bar: 50 μm). **g** Fluorescent microscopy comparing αSMA (red) expression in control (top) vs. 6g8-treated (bottom) cscTCs. Anti-DEspR- reduced αSMA-expression in Panc1-cscTCs (14.4 ± 4.3%); non-treated controls (75.2 ± 13.7%); 6g8: *n* = 7 high-power-fields (HPFs); control: *n* = 8 HPFs, ≥40 cells/HPF, *p* < 0.0001 two-tailed t-test. **h** Anti-DEspR mAbs reduced Panc1-CSC Col1A1- secretion [6g8-treated: 97 ± 16 pg/ml, 7c5-treated: 54.5 ± 2.5 pg/ml, control: 487 ± 52 pg/ml]; *n* = 4 replicate wells/group, ***p < 0.001, ANOVA multiple-comparisons test. **i** Representative double-immunofluorescence of Panc1-CDX PPC tumor: human-specific DEspR+(red), human/rat Col1A1 + (green) cscTCs within TC-islands, DEspR+Col1A1+ co-expressing cscTCs (yellow), and DEspR[−]/Col1A1+ cscTCs and stromal cells (green) in merged panel. **j **Representative Masson-Trichrome (MT)-stained **[LEFT, MIDDLE]** and H&E-stained **[RIGHT]** sections of PPC tumors, showing collagen-deposition (MT:blue, H&E:bright pink) surrounding cscTCs. Bar: 50 μm

Anti-DEspR murine monoclonal antibodies, 7c5, 5g12, and 6g8, were custom produced by ProMab (Richmond, CA) using a peptide spanning epitope 1 (7c5, 5g12) and another peptide spanning epitope 2 (6g8) of DEspR (Fig. 1a). Humanized anti-DEspR hu-6g8 was designed from the murine precursor 6g8-sequence with minimization of known T-cell epitopes and post-translational destabilizing motifs, and produced as recombinant antibody with a human hinge-stabilized IgG4^S228P^ backbone in HEK 293 pooled transient transfectants, then subsequently transferred to CHO pooled transient transfectants (LakePharma is in San Carlos, CA).

### Antibody labeling

7c5, mouse IgG2b-isotype, hu-, and human IgG4-isotype antibodies were labeled using Alexa Fluor (AF)-488 or − 568 Antibody Labeling Kit (Thermo Scientific, A20181, A20184) following manufacturer instructions.

### Collagen-1-α1 Immunostaining

Immunofluorescence staining was performed as previously described [11, 18]. Panc1 TCs and CSCs were fixed with 2% paraformaldehyde (Thermo Scientific, AAJ19943K2) for 15-min at 4 °C. Double immunostaining was done using 10 μg/ml hu--AF568, αSMA (Sigma, A5228), and collagen 1/3 alpha-1 chain-AF488 (Santa Cruz, sc-293,182, human/rat reactive), incubated overnight in 4 °C. Imaging was performed with a Zeiss Axioskop fluorescence microscope, as previously described [11, 18].

### Collagen-1-α1 ELISA

Detection of secreted COL1A1 present in Col1/3 was determined using COL1A1-ELISA kit (CusaBio Technology, CSB-E13445h) following manufacturer’s specifications. Col1A1-secretion was measured from supernatants of 7c5-treated and control Panc1 CSCs. Optical density from the assay and standards were collected at 450 nm with corrections subtracted from 540 nm, using a SpectraMax M3 microplate reader (Molecular Devices, San Jose, CA).

### Flow cytometry

Cell surface expression of Panc1 TCs and CSCs was performed as previously described [18]. Cells were labeled at 4 °C in 2% Fetal Bovine Serum (FBS) (Thermo Scientific, 10,438,026) in Hank’s Buffered Salt Solution (HBSS) (ThermoFisher, 14,170,112) for 30-min. The following antibodies were used: 10 μg/ml 7c5-AF568, 7c5-isotype control murine IgG2b-AF568 (R&D Systems, MAB0042), and/or anti-CD133-AF488 (Creative Biomart, NAB-2017-VHH). For analysis of aldehyde dehydrogenase A1 (ALDH1) activity characteristic of CSCs, we used the AldeFluor Kit (Stem Cell Technologies, 01700), and DEAB, N,N-diethylaminobenzalde-hyde, as control per manufacturer’s specifications.

For DEspR/ADAR1 flow cytometry, cells were fixed with 2% paraformaldehyde (Thermo Scientific, AAJ19943K2) then permeabilized with 0.1% Triton X-100 (Sigma, T8787) for 15-min at 4 °C respectively, then labeled at 4 °C × 20 min. The following antibodies were used: 10 μg/ml anti-DEspR hu-6g8-AF568 and anti-ADAR1 antibody (Abcam, ab126745). The anti-ADAR1 antibody was detected with an anti-rabbit IgG AF647 (Abcam, ab150079) secondary antibody. Flow cytometry was performed on an LSRII Flow Cytometer (BD Bioscience, San Jose, CA) using identical photomultiplier tube voltage settings. Further specifications are listed in Supplementary Methods (see Additional file 1).

### CRISPR/Cas9 ADAR1 knockout-out

ADAR1 CRISPR/Cas9 knockout (SantaCruz, sc-401,611) was performed following manufacturer instructions for selective knockout in Panc1 and MiaPaCa2 cell lines (see Additional file 1).

### 7c5-AF568 immunofluorescence staining

Immunofluorescence staining was performed as previously described [11, 18]. Binding was performed at 4 °C with 10 μg/ml 7c5-AF568. Internalization was induced with pre-warmed 37 °C cell media. Cells were then fixed after 15-min, 30-min, 1-h, and 2-h as noted above. Images were performed on a Zeiss Axioskop fluorescence microscope and Leica SP5 confocal microscope. Additional information is listed in Supplementary Methods (see Additional file 1).

### Hu-6g8-AF568 live-cell imaging

Panc1 were seeded onto 35 mm No. 1.5 Coverslip poly-D-lysine coated plates (Mattek, P35GC-1.5-14-C). Binding was performed at 4 °C with 10 μg/ml hu6g8AF568. The nucleus was labeled using NucBlue Live Ready Probe Reagent (ThermoFisher, R37605). Live Cell Imaging Solution (Invitrogen, A14291DJ) was used during image acquisition, and was pre-warmed to 37 °C to induce antibody internalization. Imaging was performed on an LSM 710-Live Duo Scan confocal microscope (Carl Zeiss, White Plaines, NY) with humidified (37 °C, 5% CO_2_) Pecon stage-top incubation system. Additional information is listed in Supplementary Methods (see Additional file 1).

### Hu-6g8 immunofluorescence

Panc1 or MiaPaCa2 were cultured under live-cell conditions above. For internalization studies, binding was performed at 4 °C with 10 μg/ml hu-6g8-AF568. For ADAR1 studies, Panc1 and MiaPaCa2 wild-type and ADAR1-knockout (KO) TCs were fixed and permeabilized as described above, then blocked with 1 ml of 5% bovine serum albumin (BSA) (Sigma, A7638) in Phosphate Buffered Saline (PBS) (Gibco, 13,190,144) at 4 °C for 2 h. TCs were labeled with 10 μg/ml anti-ADAR1 and hu-6g8-AF568 antibodies, followed by 2 μg/ml anti-rabbit IgG-AF488 labeled, targeting the anti-ADAR1 antibody, for 30 min at 4 °C respectively. For galectin-DEspR colocalization studies, Panc1 and MiaPaCa2 TCs were treated with 10 μg/ml of hu-6g8 for 15 min, 30 min, 2 h and 4 h with above internalization protocol. Cells were fixed and permeabilized as described above. Cells were blocked as described above. The cells were labeled with 2 μg/ml anti-human IgG-AF546 to detect internalized hu-6g8/DEspR complexes, 10 μg/ml anti-galectin-1 (gal1)-AF647 (Abcam, ab203327) and anti-galectin-3 (gal3)-AF488 (Abcam, ab207357). Nuclear detection was achieved with NucBlue, with incubation for 20 min. All confocal images were analyzed with ImageJ software (NIH). Colocalization analysis was performed using JACoP plug-in. Additional information is listed in Supplemental Methods.

### CSC tumorsphere assay

Tumorsphere assay was modified based on previously described protocols [11]. Panc1-WT and ADAR1-KO TCs, as well as MiaPaCa2-WT and ADAR1-KO TCs (p5) were plated on 96-well cell culture ultralow adherence plates, in tumorsphere media, at 250, 500, and 1000 cells/well. Cells were imaged using Celigo Imaging Cytometer (Nexcelom, Lawrence, MA), with tumorsphere colonies counted as ≥50 μm in diameter, using the Celigo tumorsphere counting protocol. A graphical representation of the experimental design is provided in Figure S2b.

### Western blot

Panc1-WT and -KO and MiaPaCa2-WT and -KO protein lysates were prepared at p5; total protein lysates were obtained using 1x Laemmli buffer (BioRad, 1,610,737) at 4 °C under gentle agitation, followed by repeated sonication and collection of lysate by centrifugation. Protein levels were assessed by absorbance at 280 nm with nucleic acid correction, using NanoDrop™ One Spectrophotometer, (Fisher Scientific, 13–400-518). Equivalent (10 μg) protein was loaded onto 4–15% Tris HCL Protein Gels (BioRad, 4,561,086). Immuno-Blot PVDF membranes were used for transfer (BioRad, 1,620,174). Blots were blocked with 5% BSA for 2-h at 4 °C. Anti-GAPDH (Abcam, ab9485, 1:2500) and anti-β-actin (Abcam, ab8227, 1:2500) were used as protein loading controls. Protein levels were assessed using hu-6g8 (25μg/ml), anti-ADAR1 (1:2000), anti-Mcl-1 (Santa Cruz, sc-12,756, 1:200), anti-human IgG HRP (Sigma AP112P, 1:10000), anti-rabbit IgG HRP (Abcam, ab6721, 1:10000), and anti-mouse IgG HRP (Abcam, ab6721, 1:10000). Antibodies were incubated for 14 h at 4 °C. Blots were developed using SuperSignal West Pico Plus chemiluminescent substrate (Fisher Scientific, 34,580), and ImageQuant LAS 4000 biomolecular imager (GE Healthcare, Chicago, IL). Densometry analysis was performed in ImageJ software, with normalization to protein-loading controls.

### 3D hu-6g8 modeling

3-D modeling of hu-6g8 was done using the ABodyBuilder tool in Therapeutic Antibody Profiler (UK). Heavy and light chains were numbered using the IMGT, Chothia, Kabat, North/Aho, and Contact numbering scheme via “Antigen receptor Numbering and Receptor Classification” ANARCI tool. Then “ABodyBuilder” was used to create a homology model of antibody sequence using SAbDab to find framework templates, FREAD to homology model loops, MODELLER/SPHINX if FREAD fails, and PEARS to model side chains [http://opig.stats.ox.ac.uk/webapps/newsabdab/sabpred/tap].

### Binding saturation

Binding saturation was performed as previously described [11]. Direct antibody binding to live Panc1 TCs and CSCs was evaluated by flow cytometry using hu-6g8-AF568 or 6g8-AF568 in sequential, serial concentrations (0.3-30 μg/ml), under conditions identical to those described above. Each data point was performed in duplicate. Summary of data is provided in Table S2.

### CSC growth inhibition

CSC inhibition was performed as previously described [11, 18], comparing hu-6g8 or in sequential, serial concentrations (0.3–30 μg/ml) using Panc1 CSCs. Panc1 CSCs were grown in 96-well non-adherent plates, and treated with hu-6g8 or 6g8 at seeding, day-2, and day-4. Live and dead CSCs were counted using Trypan Blue on day-5.

### Angiogenesis assay

HUVEC assays were performed as previously described [11], comparing hu-6g8 or 6g8 in sequential, serial concentrations (0.5–30 μg/ml), under identical conditions. Assay conditions were performed in quadruplicate. After 16-h of treatment, tube formations were digitally photographed and analyzed using ImageJ.

### Animals

Outbred Rowett nude^*nu/nu*^ (Charles River Labs) were used for all in vivo experiments. Rats were 4–5-week-old (female) or 3–4-week-old (male) at time of cell injection. All studies were performed in accordance with IACUC approved protocol. See Additional file-1 Supplemental Methods for additional information.

### Heterotopic subcutaneous Panc1 PPC model

Two-million CSCs were pretreated with 200 μg/ml or vehicle control for 1 h at 4 °C in M2 media (Sigma, M7167) prior to injection into female rats. Study ended when vehicle-control reached maximum allowable tumor size or reached 100-days (5g12). Tumor volumes were assessed at study endpoints via caliper measurements. Tumor volumes were calculated using the formula (4/3πr_1_^2^ × r_2_) where r_1_ is the larger, and r_2_ the smaller radius [11].

### Orthotopic Panc1 PPC model

For the Panc1-CDX PPC model, nude^nu/nu^ rats received an intraperitoneal injection of 200 mg/kg cyclophosphamide (Sigma, cat# C7397), 3 days prior to intraperitoneal injection of 2-million Panc1 CSCs in M2 media. For CSC pre-treatment studies, Panc1 CSCs were pretreated with 200 μg/ml 5g12 or 6g8, or vehicle control for 1 h at 4 °C. For the PPC eight-dose treatment studies, PPC-nude-rats received twice-weekly intraperitoneal injections of 1 mg/kg 6g8 or 7c5, 26 mg/kg gemcitabine (Sigma, G6423), or saline for 4-weeks, starting 7-days post-CSC injection. For the female-PPC single-dose study, PPC-nude-rats received a single-iv injection of 3 mg/kg or 15 mg/kg hu-, or a single-iv injection of 100 mg/kg intraperitoneal gemcitabine or saline.

21-days after cell injection. For the male-PPC single-dose study, PPC-nude-rats study received a single-iv injection of 15 mg/kg hu-6g8 or a single-iv injection of saline 21-days after engraftment. For neutrophil, platelet, and neutrophil-lymphocyte ratio (NLR) determinations, blood was collected at days 28, 35, and 42 post-injection in 1% EDTA. Blood was analyzed using HEMAVET 950 FS Auto Blood Analyzer (Drew Scientific, Miami Lakes, FL) with rat-species settings.

### Pharmacokinetic study

PPC rats received a single-iv bolus of 3 mg/kg or 15 mg/kg hu-6g8 4-weeks after tumor engraftment. Blood was drawn at 5-min, 15-min, 30-min, 1-h, 8-h, 24-h, 72-h, 1-wk, and 4-wks in 1% EDTA tubes, with plasma isolated by centrifugation. Protein levels were assessed using NanoDrop™ as described above, and antibody levels were measured by Western blot. Equal (10 μg) protein was loaded into 12% Mini-Protean Gels (Bio-Rad 4,561,045). Protein transfer was performed using Immuno-Blot PVDF membrane. Anti-GAPDH was used as a protein loading control under conditions identical to those above. Hu-6g8 was detected using anti-human IgG HRP (Sigma AP112P, 1:10000) for 14 h at 4 °C. Blots were developed and imaged as described above. Analysis was performed using ImageJ with densometry analysis, with concentrations determined from loaded protein standards.

### Target-engagement study

PPC models were identical to those described above. Once tumors were palpable in the greater omentum of orthotopic PPC male and female rats, they received a single-iv injection of 3 mg/kg hu-6g8 or IgG4 isotype control. After 24 h, rats were anesthetized, receiving PBS aortic perfusion prior to tissue collection under isoflurane anesthesia. PPC tumor samples and adjacent abdominal organ tissues were collected and fixed in PBS-buffered, pH 7.4, 4% paraformaldehyde. Paraffin-embedded slides were prepared (AML Labs, St. Augustine, FL), and immunofluorescence staining was performed as previously described [11]. Slides were treated overnight with 10 μg/ml AF568-anti-human IgG in a humidified chamber. Imaging was performed with a Zeiss Axioskop fluorescence microscope, as previously described [11]. To determine bioeffects, immunohistochemistry was performed using antibody to activated Caspase-3 and Ki67, with DAB secondary detection system (Mass Histology Services, Worcester, MA).

### Human tumor array

PDAC tumor tissue microarrays were obtained (US BioMax, HPan-Ade180Sur-01) as paraffin embedded tissue. Immunofluorescence staining was performed overnight with 100 μg/ml (7c5, g8) or 10 μg/ml (hu-6g8) in humidified chamber, as described above.

### Statistical analysis

Statistical analyses were performed using GraphPad PRISM 8.3 and Sigma Stat software. Paired Student’s *t-*test was used to compare means between two groups. Chi-square tests-of-independence were used to compare categorical data. Analysis of variance and non-parametric Kruskal–Wallis one-way analysis of variance were performed when appropriate for ≥3 study groups. Correlation analysis was performed using the Pearson correlation coefficient (*R*) between continuous variables. Colocalization across imaging was performed using Manders coefficient. Differences in OS were calculated with the Kaplan-Meier survival curve, Mantel-Cox log rank statistic, and Holm-Sidak multiple comparison test. *P* values were corrected using the Bonferroni multiple comparison testing. Statistically significant values were indicated as follows: *p ≤ 0.05,**p ≤ 0.01,***p ≤ 0.001, and ****p ≤ 0.0001 unless otherwise stated.

## Results

To study DEspR as a potential therapeutic target for PPC, we used multiple blocking anti-DEspR monoclonal antibodies (mAbs) raised against two extracellular domain epitopes of human DEspR (Fig. 1a). Epitope-1 anti-DEspR mAbs recognize a human-specific domain (7c5, 5g12), whereas the epitope-2 mAb, 6g8, binds to a conserved epitope in humans, rats, and monkeys, and spans the contested tryptophan [W] #14 currently annotated as a stop codon in the NCBI DNA-seq database (Fig. 1a) [18]. Both are upstream to consensus sequences for experimentally proven N-glycosylation and internalization recognition signal (IRS) consensus sequences (Fig. 1a). We assessed the efficacy of DEspR-inhibition in PPC using a stepwise, experimental system that modeled CSC-heterogeneity and plasticity, two factors which underpin cancer therapy resistance. We modeled CSC-heterogeneity by using DEspR±/CD133 ± CSCs and DEspR± TCs and cscTCs in all tests. We modeled CSC-plasticity by testing in the presence of both TC-to-CSC and CSC-to-cscTC reprogramming in in vitro and in vivo experiments. To advance a clinically-translatable therapeutic paradigm, we prioritized the study of parameters assessing anti-cancer efficacy of DEspR-inhibition at the cellular level in vitro, and subject overall survival in vivo.

### DEspR-inhibition decreases Panc1-TC and CSC stress-survival in vitro

To gain insight into efficacy, we first studied the impact of DEspR-inhibition on TC stress-survival by studying apoptotic cell morphology changes induced by anti-DEspR-mAb receptor binding and internalization. This would unify previous data reporting that DEspR cell signaling supports key pro-survival pathways [11], DEspR-inhibition decreases pro-survival gene RNA expression levels in stress-resistant Panc1-CSCs [11], and 7c5-antibody/DEspR complexes internalize and translocate to the nucleus in Panc1-TCs [18], into a putative therapeutic paradigm. We therefore characterized DEspR-bound 7c5-internalization by treating Panc1-TCs with AF568-labeled 7c5 (7c5-AF568) and tracking 7c5/receptor internalization using serial fixed-immunofluorescence microscopy. We analyzed intracellular quantity and localization of 7c5-AF568, and determined concomitant apoptotic cell morphology changes in relation to internalized 7c5-AF568.

After 1 h, we detected fluorescently-labeled AF568-7c5/DEspR internalization into the cytoplasm with some nuclear localization, in contrast to minimal non-specific internalization of the AF568-IgG2b isotype-control (Fig. 1b). By 2-h, we observed further nuclear localization and a significant increase in various apoptotic cell morphology changes in AF568-7c5-treated Panc1 TCs in contrast to the lack of nuclear localization in isotype-treated controls (Fig. 1b). A few cells in 7c5-AF568 treated TCs exhibited rounded cell swelling rather than apoptosis cell shrinkage, consistent with necroptosis phenotype; none detected in isotype controls (Fig. 1b). These data are concordant with previous data showing that overnight DEspR-inhibition using 7c5 in Panc1-CSCs decreases anti-apoptotic Mcl-1 and anti-apoptosis/necroptosis CIAP2 or BIRC3 RNA levels [11], both key pro-survival proteins in PDAC TCs [19, 20].

### DEspR-positive/negative subsets among TCs, cscTCs and CSCs

To assess DEspR-accessibility as a therapeutic target in PPC, we assessed its relative cell surface expression by flow cytometry. We compared Panc1-TCs and Panc1-CSCs as components of CSC-TC plasticity and heterogeneity [10], and tested whether DEspR cell-surface expression increases in anoikis-resistant Panc1-CSCs. To this end, we studied functionally selected anoikis-resistant CSCs from reprogrammed Panc-1-TCs regardless of marker cell-surface expression. These CSCs exhibited high tumorigenicity previously validated in vivo [11].

As shown in Fig. 1c, we detected cell-surface DEspR in more than 50% of Panc1 TCs and CSCs supporting therapeutic target accessibility. The Panc1 TCs tested comprised both DEspR+ or DEspR- cell-populations, whereas Panc1 CSCs consisted of three subsets: low-DEspR+, high-DEspR+, and DEspR- populations (Fig. 1c), validating cell heterogeneity. Panc1 CSC-populations expressed known PDAC-CSC markers, ALDH1 and CD133 [8]: ALDH1 activity in 68–72% and CD133+ expression in 28–30% of CSCs. Notably, most (25–27%) CD133+ CSCs exhibit DEspR+ expression (Fig. 1c). We also investigated DEspR cell surface expression on other PDAC cell lines: MiaPaCa2 (58–65%) and Capan-1 TCs (31–40%) (Fig. 1d). We further studied and confirmed DEspR+ in CSC-clusters by AF568-7c5 immunostaining of dissociated CSC-spheroids immobilized on a chamber glass slide (Fig. 1e). Altogether, data show DEspR expression across the CSC-plasticity spectrum spanning CSCs, cscTCs, TCs, and CSC micro-clusters, and demonstrate variations in molecular marker-based subsets (DEspR±, CD133±), thus confirming CSC-heterogeneity as seen in human PDAC [8] and other cancers [10].

### DEspR-inhibition decreases CSC anoikis-resistance and spheroid formation

To determine the impact of DEspR inhibition on CSC anoikis resistance and tumorsphere formation in the presence of different CSC-subsets, we treated DEspR± Panc1-CSCs with fluorescently-tagged AF568-7c5, then cell-sorted fluorescent DEspR+ from DEspR- CSCs by MoFlo Cell sorting. Testing survival in low adherence anoikis-culture conditions, we observed that Mo-Flo sorted DEspR[−] CSCs grew by 5 days, forming sheet-like clusters, rather than tumorspheres (Fig. 1e-third panel). However, after the 7c5-bound/sorted DEspR+ CSCs were non-viable (Fig. 1e-fourth panel). Interestingly, the subsequent passage of the DEspR[−] CSC-pool in low adherence culture conditions re-established a DEspR+ pool in 30–40% of CSCs as detected by flow cytometry. These observations confirm DEspR roles in CSC stress (anoikis)-survival which impacts stemness-associated spheroid formation.

### Detection of ACTA2 (αSMA) and Col1A1 expression on CSCs and cscTCs

To determine the effects of DEspR inhibition on Panc1-CSC and cscTC mesenchymal functions relevant to peritoneal dissemination, we assessed alpha-smooth muscle actin (αSMA) expression, and concomitant downstream expression and release of collagen-1 (Col1A1) given their prometastatic functionality and potential contritubution to desmoplasia in the tumor microenvironment. Immunofluorescence detected αSMA expression in TNF-α stimulated cscTCs (Fig. 1f), which prompted evaluation of collagen1 (Col1A1) expression, as αSMA expression in activated fibroblasts is associated with upregulation of Col1A1 expression and secretion, but TNF-α decreases Col1A1 in dermal fibroblasts. Surprisingly, αSMA+ cscTCs co-expressed Col1A1 in pure cscTC cultures (Fig. 1f) without fibroblast co-cultures.

To examine the impact of DEspR-inhibition on αSMA/Col1A1 expression, we tested anti-DEspR 6g8- and 7c5-mediated DEspR inhibition in Panc1-cscTCs and CSCs. Anti-DEspR 6g8 treatment suppressed both the expression of αSMA in cscTCs (Fig. 1g) and the expression/secretion of Col1A1 from CSCs in vitro (Fig. 1h). Similarly, anti-DEspR 7c5 suppressed Col1A1 expression/secretion from CSCs (Fig. 1h). Whether this suppression is direct or via induction of apoptosis-associated function-shutdown remains to be determined.

To study DEspR-Col1A1 co-expression in vivo, we examined tumor sections from Panc1-CSC derived xenograft (CDX)-PPC nude rats by immunofluorescence (IF) using human-specific DEspR (7c5) and human/rat reactive Col1A1 fluorescently labeled antibodies. Multiplex-IF shows that PPC-TCs in tumor cell islands co-express human-specific DEspR and Col1A1, but that DEspR−/Col1A1+ TCs are also present in the same tumor cell islands (Fig. 1i). Human-specific DEspR+/Col1A1 Panc1-CSC-derived TCs in PPC tumor sections are distinguished from rat host stromal fibroblasts by cell morphology, location, and negativity for human-DEspR+ expression (Fig. 1i). Concordantly, representative Masson Trichrome and H&E-stained section (Fig. 1j) shows pericellular collagen deposition. These data demonstrate that Panc1 cscTCs and CSCs also contribute to peri-cellular collagen deposition in PPC tumors.

### ADAR1 regulation of DEspR protein in Panc1 and MiaPaCa2 nonCSC-TCs

To address the discordant NCBI annotation of the DEspR locus as a transcribed non-coding gene, FBXW7-antisense RNA (AS-1), with our data showing DEspR protein expression and functionality [18], we tested whether ADAR1 RNA-editing would reconcile this discrepancy via A/I(G) RNA editing. Since site-specific RNA editing can occur at levels as low as 0.1%, with the average being only 20% RNA-edited species [21], a low, but physiologically relevant, percentage of RNA-edited transcripts could be vulnerable to algorithm-based exclusion as ‘noise/error’ in high-throughput RNA-seq databases. To affirm that DEspR meets the structural requirements for ADAR1 binding, DNA database analysis shows that the DEspR RNA spanning the contested tryp-#14 exists as double-strand-RNA (dsRNA) with the FBXW7-RNA on the antisense strand, thus meeting the dsRNA-requirement for ADAR1-binding for RNA-editing [21]. Furthermore, the DEspR-RNA sequence spanning the contested RNA-edited A/I(G) site contains a putative hairpin loop with three smaller loops that is concordant with secondary structure requirements sufficient to guide ADAR1 binding for nucleotide-specific editing [22] (Fig. S1a).

**Fig. 2:**
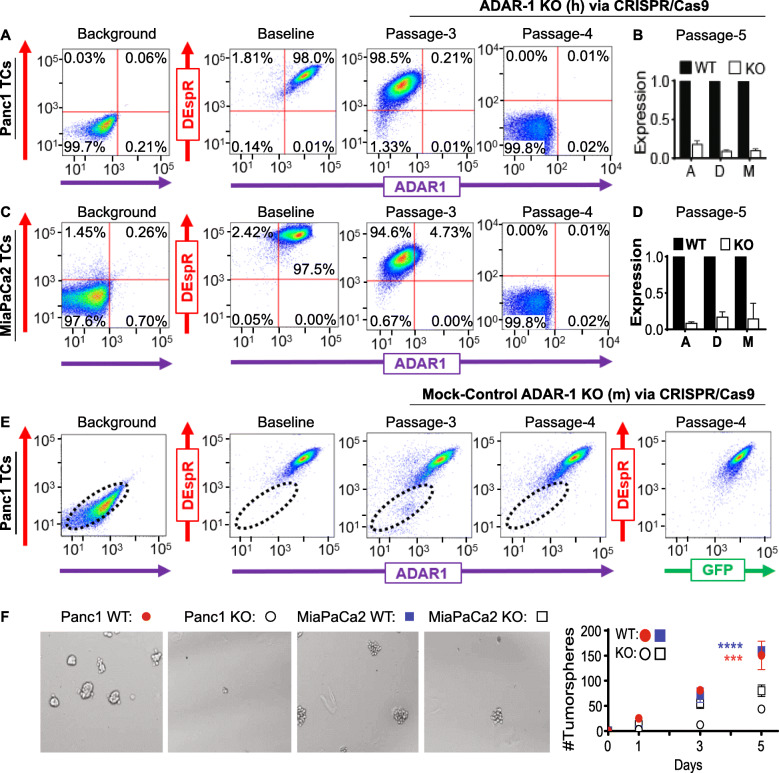
ADAR1 expression is required for DEspR protein expression in Panc1 and MiaPaCA2 tumor cells (TCs). **a** Flow cytometry analysis of permeabilized Panc1-TCs showing baseline 98% ADAR1+/DEspR+ co-expression. CRISPR/Cas9-knockout (KO) TCs at passage (p)-3 exhibited 0.22% ADAR1+, 98% DEspR+ expression; at p4: 0.01% DEspR+/ADAR1+ (100,000 cells/duplicate); see Table S1. **b** Quantitative western-blot analyses of p5 ADAR1-KO Panc1-TCs showing decreased ADAR1(A) (18.5% ± 4.1%), DEspR(D) (9.4% ± 1.3%), and Mcl-1(M) (10.4% ± 1.8%) compared to wild type (WT) Panc1-TCs (100%-reference). [Western blots in Fig. S3]. **c** Flow cytometry study of permeabilized MiaPaCa2-TCs showing 97.5% baseline co-expression of ADAR1+/DEspR+. CRISPR/Cas9-ADAR1-KO TCs had 4.73% ADAR1 + expression (94.6% DEspR+) by p3; 0.02% DEspR+ADAR1+ at p4 (100,000 cells/*n* = 2); Table S1. **d** Quantitative western-blot analyses of ADAR1-KO MiaPaCa2 TCs showing decreased ADAR1(A) (9.0% ± 1.3%), DEspR(D) (17.5% ± 6.4%), and Mcl-1(M) (32.9% ± 5.6%) compared to WT MiaPaCa2-TCs (100%-reference). [Western blots in Fig. S3] **e** Mock-control for ADAR-1 KO using murine-specific CRISPR/Cas9-ADAR1-KO construct showing no knockout of human-ADAR1 and no loss of DEspR expression by p4 despite successful transfection (reporter-GFP+ expression). **f** Representative phase-contrast images at 5-days post-seeding showing decreased survival/growth and tumorsphere-formation in low adherence conditions of p5 ADAR1-KO vs WT Panc1 and MiaPaCa2 TCs. Graph of number(#) of tumorspheres > 50 μm-diameter on day-1, − 3, and − 5 post-seeding comparing ADAR1-WT and ADAR1-KO tumor cells (***p = 0.000036) and MiaPaCa2-TCs (****p = 0.000002); paired two-tailed t-test, Bonferroni-Dunn correction, 5-replicates/point

To obtain experimental evidence for ADAR1-dependent DEspR expression, we first performed double-immunostaining of two PDAC-TC lines, Panc1 [KRAS^G12D^] and MiaPaCa2[KRAS^G13D^] to determine if every ADAR1-expressing TC also expresses DEspR. Data show that ADAR1 and DEspR co-expression overlap in both Panc1-TCs and MiaPaCa2-TCs, such that all DEspR+ TCs expressed ADAR1 (Fig. S1b-c). Next, we performed ADAR1-knockout using a CRISPR/Cas9 knockout system (Fig. S2a). To determine the effects of ADAR1-knockout, we performed a timed-series flow cytometry analysis. This confirmed co-expression of DEspR and ADAR1 proteins in DEspR+ cells in Panc1 (Fig. 2a) and MiaPaCa2 (Fig. 2c) TCs at baseline, loss of ADAR1 expression by 3rd passage after transfection, and subsequent loss of DEspR-expression in both Panc1 (Fig. 2a) and MiaPaCa2 (Fig. 2c) by the 4th passage (Table S1). Mock-control knockout of Panc1 and MiaPaCa2 cells using the mouse-specific ADAR1 construct did not reduce human-ADAR1 nor human-DEspR expression, but confirmed transfection via GFP-reporter gene expression (Fig. 2e), thus affirming the specificity of human ADAR1 knockout experiments (Fig. 2a, c), and ruling out confounders from transfection process and/or from puromycin-based selection.

Western blot analyses of passage-5 (p5) ADAR1-KO Panc1- and MiaPaCa2-TCs confirmed decreased ADAR1 and DEspR protein levels, and detected decreased Mcl-1 (Fig. 2b, d, Fig. S1d-f), a key pro-survival protein in PDAC [19]. Functionally, loss of ADAR1+/DEspR+ TC-subsets led to decreased anoikis resistance in low adherence cultures and decreased spheroid formation, a known marker of stemness (Fig. 2f) in mixed-pool Panc1- and MiaPaCa2-TCs. These data confirm anti-DEspR 7c5-mediated decreases in Panc1-CSC tumorsphere-formation (Fig. 1) and Mcl-1 transcript levels [11].

### Improved functionality of recombinant humanized anti-DEspR mAb, hu-6g8

**Fig. 3:**
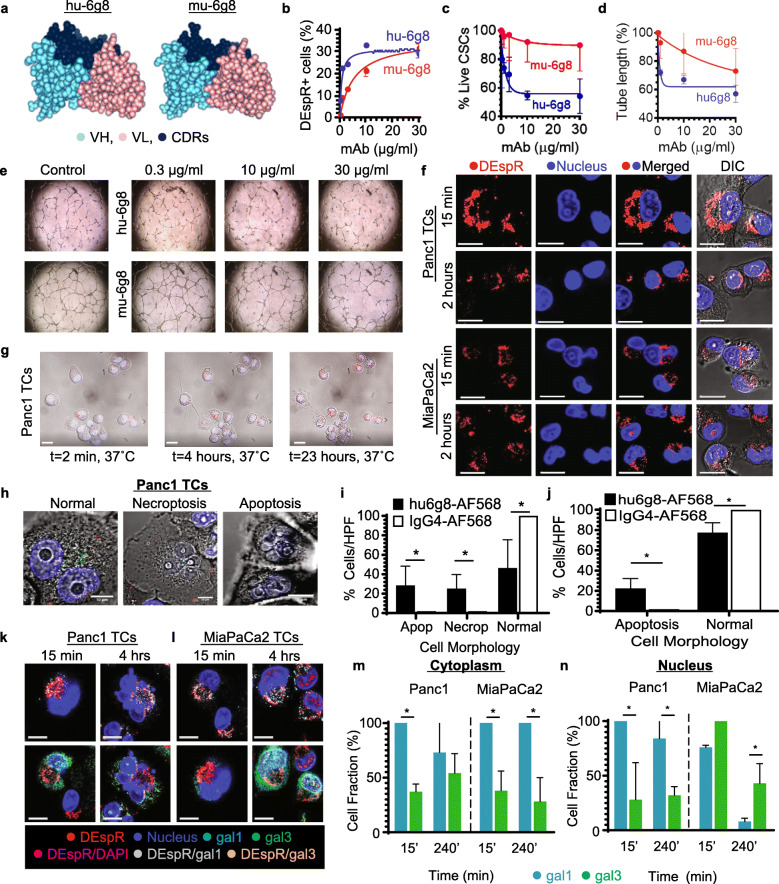
Humanized anti-DEspR mAb exhibits improved potency, retains DEspR/mAb internalization and nuclear translocation. **a** 3D-model of humanized anti-DEspR **[Left]** hu-6g8, **[Right]** mu-6g8: complementary determining regions (navy), heavy-chain (aqua), light-chain (pink). **b**-**d** Comparison of hu-6g8 and mu-6g8 **b** binding-affinity to intact DEspR on Panc1-TCs, **c** inhibition of Panc1-CSCs, and **d** HUVECs angiogenesis (EC_50_, IC_50_ values: Table S2). **e** Representative images of hu-6g8 and mu-6g8 angiogenesis inhibition, showing concentration-dependent decreased HUVEC tube-formation. **f** Confocal immunofluorescence of hu-6g8/DEspR internalization and nuclear-translocation (t = 15-min to 2-h) in Panc1 and MiaPaCa2 TCs. Bar = 15 μm. **g** Live-cell imaging of apoptotic changes in hu-6g8-treated Panc1-TCs. Bar = 20 μm. **h** Higher-magnification live-cell images of hu-6g8-treated Panc1 TCs showing normal, necroptotic, and apoptotic cell morphology. Bar = 10 μm. **i**, **j** Quantitative analysis of apoptotic (apop) and necroptotic (necrop) morphological changes in hu-6g8-treated. **i** Panc1-TCs (*n* = 208, *p* < 0.0001) and **j** MiaPaCa2 (*n* = 284, *p* < 0.0001) vs. isotype-control at 2 h; chi-squared test for independence, paired t-test. **k**, **l** Internalization of hu-6g8/DEspR complexes detected by AF568-labeled anti-human-IgG mAb (red), and colocalization with cytoplasmic-nuclear shuttling proteins: galectin-1 (gal1[aqua]) or galectin-3 (gal3[green]) in **k** Panc1 and **l** MiaPaCa2 TCs, after 15-min, and 4-h of hu-6g8-treatment. Bar = 20 μm. **m**, **n** Colocalization of DEspR/gal1(aqua) or DEspR/gal3(green) (Mander’s coefficient κ > 0.5) in Panc1 and MiaPaCa2 TCs in the cytoplasm (**m**) or nucleus (**n**), at 15-min and 4-h (Table S3). (*p < 0.05, *n* = 261 and 251 TCs, Panc1 and MiaPaCa2, respectively, paired two-tail t-test)

To test the translatability of DEspR-inhibition, we developed and characterized a recombinant, humanized anti-DEspR mAb using the antibody sequence of 6g8, selected for its conserved epitope sequence in human, rat, mouse, and non-human primates (Fig. 1a). We designed the humanized antibody with a hinge-stabilized S228P human-IgG4 backbone, reduced sequence-motifs of T-cell epitopes, and post-translational destabilization in silico, and produced it as a recombinant mAb in a transient HEK293 expression system. Comparison of 3-D modeling of hu-6g8 and its precursor, 6g8, demonstrated greater potential surface-exposure of the complementarity determining regions (CDRs) in hu-6g8 (Fig. 3a). We demonstrated that hu-6g8 exhibits improved binding to live Panc1-TCs (Fig. 3b, Fig. S4a), improved inhibition of Panc1-CSC survival in anoikis conditions (Fig. 3c), and had improved anti-angiogenic activity (Fig. 3d, e), with greater potency compared to 6g8 (Table S2).

### Hu-6g8 internalization, nuclear translocation, and apoptosis effects

Next, we studied whether hu-6g8 demonstrates similar internalization kinetics and cell-survival inhibition to murine-7c5. We tracked hu-6g8 internalization via time-stopped series of confocal microscopy in Panc1- and MiaPaCa2-TCs treated with unlabeled hu-6g8 and stained with anti-human IgG-AF568. Significant intracellular hu-6g8/DEspR complex accumulation occurred by 15 min (Fig. 3f, Fig. S3a), similar to 7c5-treatment. We observed intracellular accumulation of hu-6g8/DEspR similar to 7c5/DEspR, with translocation towards and into the nucleus by 1-h in both Panc1 and MiaPaCa2 TCs, and associated apoptotic nuclear changes by 2 h (Fig. 3f). Live cell imaging corroborates hu-6g8/DEspR internalization and associated apoptotic cellular changes (Fig. 3g-h). Quantitative analysis of cellular morphologies (Fig. 3h) detected significant induction of apoptosis by hu-6g8 in both Panc1 and MiaPaCa2 TCs in these experimental conditions compared with isotype-treated TCs (Fig. 3i, j). Necroptotic swelling was also present in Panc1 (Fig. 3i) but not in MiaPaCa2 TCs (Fig. 3j).

Internalization of both 7c5/DEspR and hu-6g8/DEspR is concordant with a consensus IRS in DEspR’s protein sequence (Fig. 1a). However, as DEspR has no canonical nuclear transport signal, we investigated direct nuclear shuttling via galectin-1 (gal1) and galectin-3 (gal3) proteins capable of two-way nucleo-cytoplasmic shuttling [23, 24]. Both gal1 and gal3 proteins were identified as associated with glycosylated DEspR in DEspR-targeted pulldown experiments [18]. In fixed-cell confocal imaging, we tracked and detected hu-6g8/DEspR complex internalization and colocalization with gal1 or gal3 via multiplex immunostaining from 15 min to 4 h, and rigorously confirmed nuclear localization across Z-stack images (Fig. S3b). We detected co-occurrence of the internalized hu-6g8/DEspR complex with gal1 and gal3 in Panc1-TCs (Fig. 3k) and MiaPaCa2-TCs (Fig. 3l) after 15 min to varying degrees (Fig. S3b), which was not observed in isotype controls. We also observed apoptotic nuclear morphological changes across 4 h (Fig. 3k, l), consistent with prior live cell imaging findings (Fig. 3i, j).

Quantitative analysis of colocalization of hu-6g8/DEspR with gal1 and/or gal3, defined by Manders’ overlap coefficient κ > 0.5 (Fig. S3d, Table S3), corroborated observed co-occurrence of hu-6g8/DEspR immunostaining with both gal1 and gal3 in the cytoplasm and nucleus (Fig. 3k-n). Interestingly, hu-6g8/DEspR complexes associated with gal1 more than gal3 in both Panc1 and MiaPaCa2 TCs in the cytoplasm (Fig. 3m, Table S3). Panc1-TCs exhibited higher hu-6g8/DEspR-gal1 nuclear colocalization than hu-6g8/DEspR-gal3, while MicaPaCa2-TCs exhibited the inverse (Fig. 3n, Table S3). Additionally, the proportion of MiaPaCa2 TCs with nuclear hu-6g8/DEspR-gal1 colocalization decreased significantly from 15-min to 4-h and was associated with characteristic apoptosis nuclear changes (Fig. 3l, n, Fig. S3b).

### DEspR-inhibition of Panc1-CSC tumorigenicity and transperitoneal dissemination

**Fig. 4:**
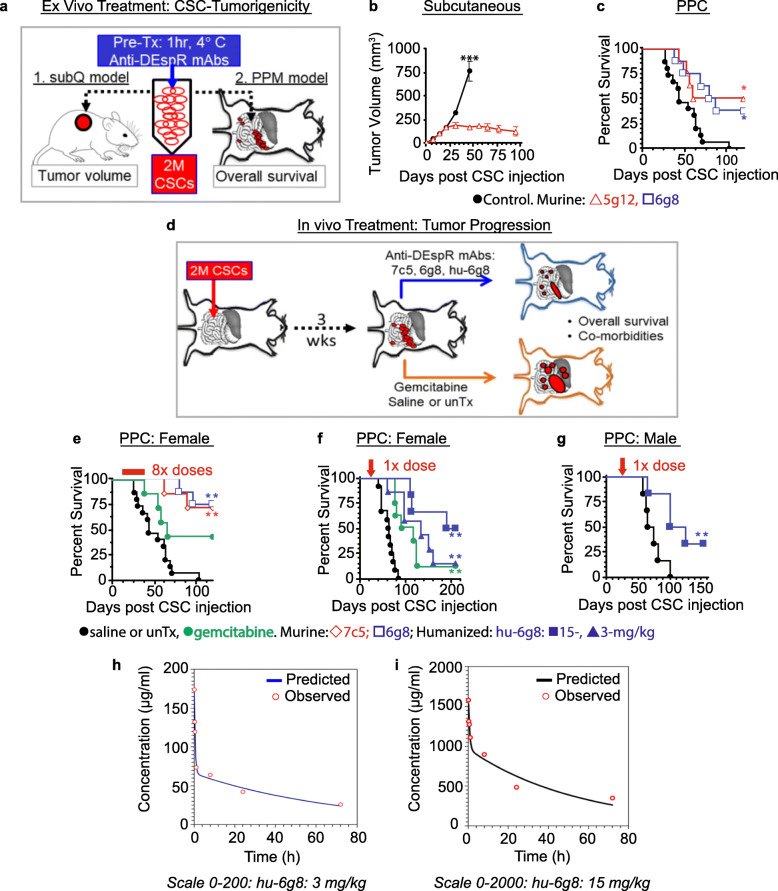
Epitope-distinct murine and humanized anti-DEspR mAbs inhibit PPC dissemination-progression. **a** Diagram of anti-DEspR ex vivo pre-treatment design evaluating CSC tumorigenicity in Panc1-CSC subcutaneous and PPC xenograft nude-rat models. **b** Ex vivo pre-treatment with 5g12 (red) 200 μg/mL × 1-h (*n* = 6) decreased Panc1-CSC tumorigenicity vs saline controls (*n* = 8) (*p* < 0.001, repeated t-test. **c** Ex vivo pre-treatment of Panc1-CSCs with anti-DEspR 5g12 (red, *n* = 8) and 6g8 (blue/*n* = 8) (200 μg/ml × 1-h) increased survival vs saline control (black/*n* = 15) (*p* = 0.0088, log rank test). **d** Diagram of in vivo anti-DEspR treatment in PPC nude-rat models. **e** Survival analysis: PPC-females comparing saline-control (*n* = 15) vs multi-dose (1 mg/kg ip 2x/wk. × 4 wks) murine anti-DEspR epitope-2 6g8 (*n* = 8, *p* = 0.0002), epitope-1 7c5 (*n* = 7, *p* = 0.002), and gemcitabine 26 mg/kg (*n* = 7, ns). **f** Survival analysis: single-dose-treated PPC-females 3 wks after CSC-ip injection: saline controls (*n* = 12), hu-6g8 at 3 mg/kg (*n* = 8, *p* = 0.001) or 15 mg/kg (*n* = 7, *p* = 0.0007), or gemcitabine 100 mg/kg (*n* = 7, *p* = 0.0018). **g** Survival analysis in single-dosed PPC-males: hu-6g8 15 mg/kg/dose (*n* = 6) vs saline (*n* = 6, *p* = 0.02). Kaplan-Meier survival analyses with log rank and Holm-Sidak multiple pairwise comparisons testing (Table S4). **h**, **i** Single-dose hu-6g8 pharmacokinetic analysis in PPC-females at t-21 days to match tumor burden and treatment onset to survival studies. Two-compartment analyses at intravenous-dosing: 3 mg/kg (**h**) and 15 mg/kg (**i**). T_1/2_,_avg_ = 46.1 h, maximum retention time (MRT_avg_) = 65.2 h

Having demonstrated efficacy of DEspR-inhibition of CSC/TC stress-survival and functionalities in vitro, we next tested whether DEspR-inhibition of mixed DEspR± Panc1-CSCs would suffice to reduce tumorigenicity in vivo, given targeted-sparing of all DEspR[−] CSCs. We used a heterotopic-subcutaneous xenograft model in nude rats (Fig. 4a) to attain 10x larger and hence, more complex and heterogeneous tumors than would be attainable in mice, and to facilitate tracking tumor volume and invasiveness [11]. We observed that one-hour treatment of DEspR± Panc1-CSCs with human-specific epitope-1 5g12-mAb prior to subcutaneous injection decreased tumorigenicity as measured by tumor volume, a net decrease that was sustained over time compared to non-treated Panc1-CSCs (Fig. 4b). Similarly, 1-h pre-treatment of mixed DEspR± Panc1-CSCs prior to intraperitoneal injection (Fig. 4a), using epitope-1 (5g12) and epitope-2 (6g8) mAbs (Fig. 1a), decreased Panc1-CSC peritoneal dissemination, resulting in significantly increased survival of PPC-rats compared to non-treated CSC-controls (Fig. 4c). Since 7c5 and 5g12 are human-DEspR-specific, and CSC mixed-pools comprise both DEspR+ and DEspR- CSCs, net-decreased tumorigenicity reflects the importance of DEspR+ CSC subset in tumor establishment, dissemination, and progression. The more robust impact on tumorigenicity in subcutaneous vs peritoneal xenografting is concordant with greater metastatic-permissiveness in the peritoneal microenvironment, as observed in PDAC patients [25].

To test the impact of anti-DEspR therapy on overall survival (OS) as a translatable and clinically relevant endpoint, we used a Panc1-CDX-PPC nude rat model (Fig. 4d). This Panc1-CDX-PPC model recapitulates key clinical PPC features and predilection for the greater omentum [26], along with key clinical comorbidities including: ascites, jaundice, gut dysfunction, and high mortality [27]. We excluded primary PDAC to attain a timed onset of PPC to enhance reproducibility of survival studies which a priori requires quantitatively equivalent onset and tumor burden for each study subject. Additionally, a PPC-only model can determine the impact of PPC per se on overall survival and comorbidities, as well as test CSC capability in peritoneal dissemination-progression without need for pre-metastatic niche formation [28].

Treatment started 7-days after Panc1-CSC intraperitoneal (ip) injection, a time point when PPC rats exhibit multiple (> 20) visible (1–3 mm) peritoneal tumors, which in patients would contraindicate curative-intent surgery. Murine mAbs 7c5- and -treatments significantly increased OS compared to saline mock-treated controls (Fig. 4e, Table S4). In contrast, standard-of-care gemcitabine (26 mg/kg/dose ip), equivalent to mouse dose 198 mg/kg (see Additional file 1: Supplementary Methods), or human dose 1000 mg/m^2^ did not significantly extend OS compared to saline-control rats (Fig. 4e, Table S4).

Next, we tested the humanized clinical candidate hu-6g8 in PPC nude rats at a more advanced stage, approximately 3–4 weeks post-Panc1-CSC intraperitoneal xenografting (Fig. 4d), recording both efficacy and adverse events. We compared hu-, at 3-and 15-mg/kg/dose given intravenously (iv), to gemcitabine at 100-mg/kg/dose intraperitoneally (ip), equivalent to ~3x-human 1000-mg/m^2^ dose, or 760 mg/kg mouse dose (see Additional file 1: Supplementary Methods). At this stage, PPC rats typically exhibit multiple palpable peritoneal tumors (> 20 tumors: ≥ 5-mm diameter), at times in matted confluence, and with dissemination to the other abdominal organs and retroperitoneal space, but prior to comorbid ascites, jaundice, or intestinal dysfunction. We used single-dose treatment regimens to eliminate confounders from rat-host immunogenic response to foreign/human IgG4-protein, and used single high-dose 100 mg/kg ip gemcitabine with dose-limiting toxicities if given 2 doses. Notably, single-dose hu-6g8 extended median survival significantly in a dose-dependent manner, compared to mock-treated saline control (Fig. 4f). Survival outcomes were equivalent between 3-mg/kg hu-6g8 and high dose gemcitabine (Fig. 4f); whereas, 15-mg/kg hu-6g8 single-dose treatments showed significant improvement in survival compared to dose-limiting gemcitabine therapy.

To assess for potential sex-dependent efficacy, we tested Panc1-CDX-PCC male rats using the identical single dose of 15-mg/kg hu-6g8, the more efficacious dose in PPC female rats. We observed a similar improvement in survival between female (Fig. 4f) and male rats (Fig. 4g) with advanced-PPC, consistent with sex-independent efficacy. We also initiated a survival study in MiaPaCa2 CDX-PPC nude rat model but aborted this survival-study due to < 100% tumor penetrance in non-treated controls despite identical conditions with Panc1-CDX-PPC model. Additionally, intraperitoneal administration of hu-6g8 mAb gave equivocal results suggesting vulnerability of hu-6g8 mAb to proteases present in advanced PPC-peritoneal/ascites fluid.

**Fig. 5:**
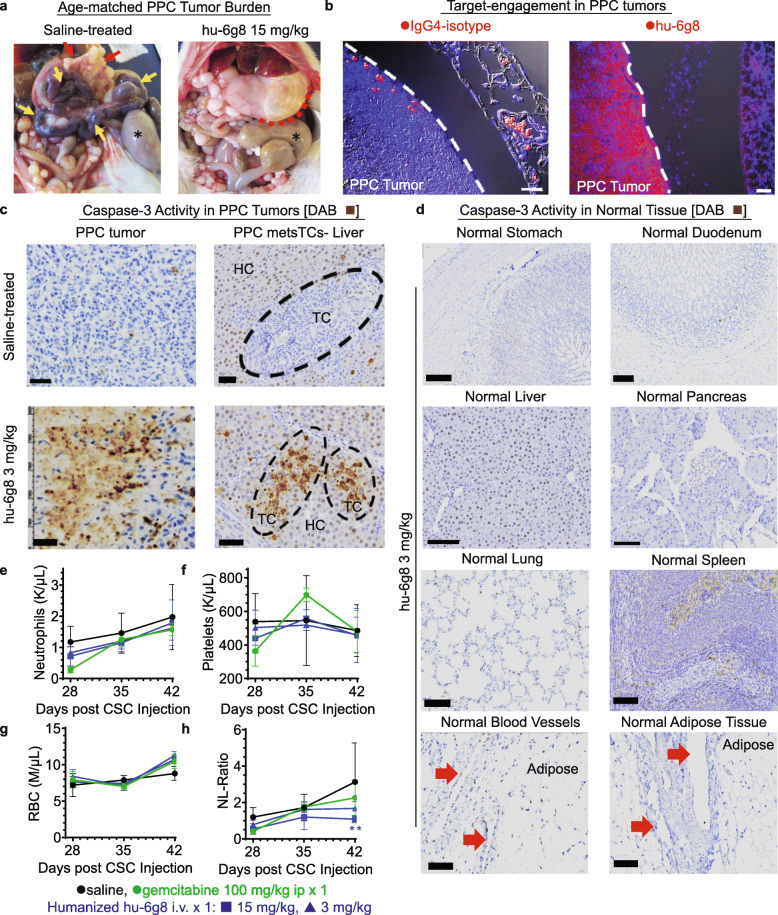
Analysis of anti-DEspR treatment effects on tumor burden, target engagement and bioeffects. **a** Representative post-mortem images of age-matched (57-days post-CSC injection) PPC saline-treated [Left] and hu-6g8 15 mg/kg-treated [Right] rats; hu- significantly decreased tumor burden. Comorbidities: dilated, necrotic small intestine in saline rats only (yellow➔), high omental tumor burden in saline (red➔) vs. minimal in hu-6g8 (**− − -**), cecum (*): dilated in saline but not in hu-6g8. **b** Target-engagement (red/magenta immunofluorescence+) 24-h after infusion on PPC tumors: [**Left**] IgG4-isotype: minimal, [**Right**] hu-6g8: high levels; DAPI+ nuclei (blue), colocalized hu-6g8/nucleus (magenta), RBC-autofluorescence (peach). **c** Target-bioeffects: activated Caspase-3 apoptosis DAB-staining (brown) in PPC tumors: [**Left**] omental tumors; [**Right**] tumor cells in liver [**Top**] saline control; [**Bottom**] hu-6g8 3 mg/kg iv. TC, PPC-tumor cells; HC, normal hepatocytes. Bar: 60-μm. **d** Representative immunohistochemistry images showing no activated Caspase-3 staining for apoptosis in normal tissues. Tumor vasculature (red➔). Bar = 100 μm. **e**-**g** Effect of hu-6g8 on hematologic cells: **e** neutrophils, **f** platelets, and **g** red blood cells (RBCs); no significant differences observed among saline, single-dose gemcitabine, and single-dose 3- and 15-mg/kg hu-6g8. **h** Significant difference (**) detected in neutrophil-lymphocyte (NL)-ratio with 15 mg/kg hu-6g8 treatment (*n* = 4) vs saline (*n* = 11), *p* = 0.009, two-way ANOVA, Bonferroni-correction

In addition to survival benefits, hu-6g8 treated CDX-PPC rats had decreased tumor burden and comorbidities compared to untreated PPC-rats. Comparing the tumor burden of saline mock-treated PPC rats at time of death to a contemporary age-matched 15-mg/kg hu-6g8-treated PPC rat that was euthanized to match tumor duration, we observed greater omental tumor burden, as well as distended gut, ascites, jaundice, and biliary obstruction from tumor invasion at the *porta hepatis* in the saline-control PPC rat, but not in the hu-6g8 treated rat (Fig. 5a).

To gain translational pharmacological insight, we next characterized the pharmacokinetics of hu-6g8 in PPC rats. Two different single-dose boluses, 3- and 15-mg/kg iv were used in PPC female rats matched for age and tumor burden with prior survival experiments (Fig. 4h, i). Hu-6g8 fit a two-compartment distribution with rapid distributional half-life (0.326–0.484 h) consistent with a high target-load due to high tumor burden present in PPC rats 3-weeks after xenografting CSC ip-injection. The relatively short half-life (~ 3 days observed in both doses), in contrast to normal human 21-day IgG4-half-life, likely reflects target-mediated clearance and interspecies differences in FcRn affinity [29].

To further assess pharmacological parameters in support of in vivo efficacy, we tested for hu-6g8 target engagement and bioeffects in CDX-PPC female and male rats with established peritoneal metastatic tumors (≥ 3 weeks after CSC ip-injections) 24-h after iv-infusion of a single 3-mg/kg dose. In contrast to human IgG4-isotype infused tumors, with minimal immunofluorescent-positive target-engagement, tumors from hu-6g8 treated PPC rats exhibited human-specific IgG immunofluorescence in the majority of TCs, indicating target-engagement, (Fig. 5b, Fig. S4b). Furthermore, we detected good tumor penetration of hu-6g8 and high tumor specificity when contrasted to normal pancreas exhibiting zero-immunofluorescence (Fig. 5b, Fig. S4b). These data support clinical feasibility as potential PPC therapy.

To test for predicted target-bioeffects 24-h after infusion, we analyzed adjacent serial sections for apoptosis measuring activated caspase-3 by immunohistochemistry (IHC). Isotype mock-treated tumors exhibited minimal activated caspase-3 in contrast to increased activated caspase-3 immuno-staining in hu-6g8-treated PPC-tumor cells (Fig. 5c). Importantly, adjacent tissues from normal stomach, duodenum, liver, pancreas, lung, spleen, vasculature, and adipose (Fig. 5d) did not exhibit induced-apoptosis in hu-6g8-treated PPC-CDXs, indicating tumor-targeted specificity and sparing of normal DEspR[−] tissues. To assess impact on tumor cell proliferation, we observed that hu-6g8-treated tumors demonstrated decreased cellular proliferation measured by number of Ki67+ tumor cells on immunohistochemistry (IHC) (Fig. S4c). We also observed decreased number of tumor microvessels in tumor areas with decreased Ki67-IHC and increased tumor cell loss (Fig. S4c).

To gain insight into safety, we investigated potential myelosuppression from anti-DEspR antibody-therapy. Treatment with hu-6g8 doses up to 15-mg/kg/dose iv did not induce neutropenia (Fig. 5e), thrombocytopenia (Fig. 5f) or anemia (Fig. 5g) in PPC-nude rats. Interestingly, anti-DEspR mAb therapy significantly reduced neutrophil-lymphocyte ratios (NLR) (Fig. 5h), a potential surrogate early PDAC response parameter [30]. Importantly, we observed no acute adverse events in female or male PPC-nude rats treated with 3- and 15-mg/kg/dose hu-6g8, nor with any of the murine mAbs 7c5, 5g12, 6g8.

### Insight into clinical translational relevance

**Fig. 6:**
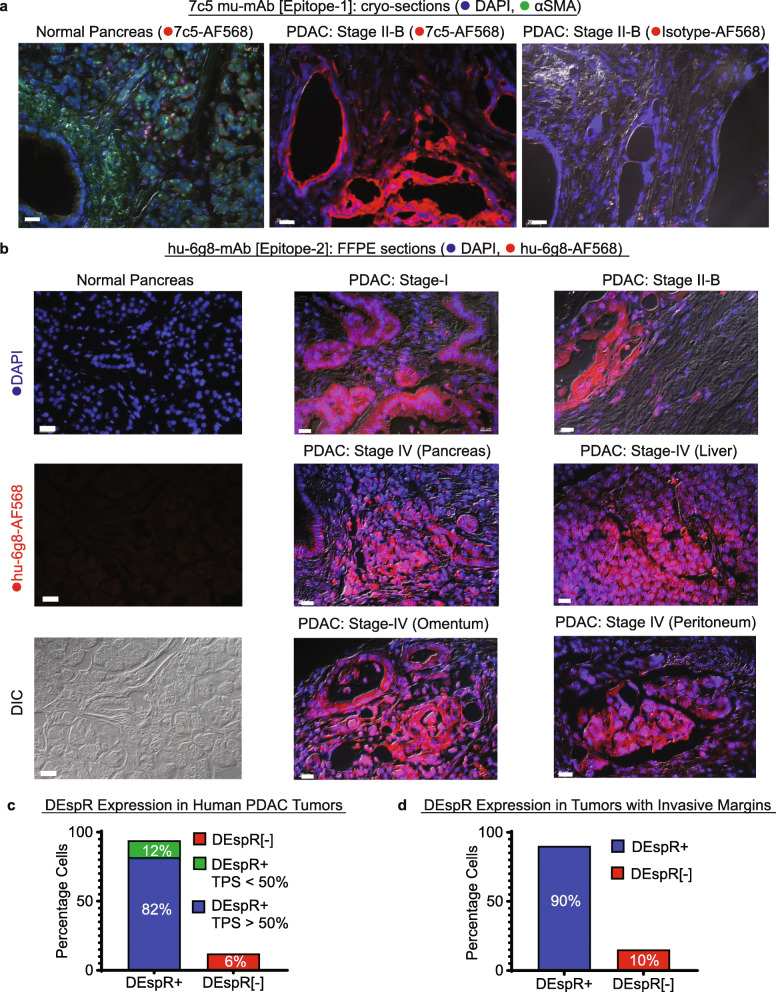
DEspR is expressed in human PDAC primary and metastatic tumors. **a** Representative immunofluorescence images of cryo-sections of normal human pancreas and human PDAC Stage-II-B tumors probed with AF568-labeled 7c5[epitope-1] murine-mAb (red) and DAPI+ nuclei (blue). Bar = 20 μm. **b** Representative immunofluorescence images of paraffin-embedded, antigen-retrieved sections of [Left] human normal pancreas and [Right] human PDAC primary tumors at different stages (I-IV), and metastatic (met.) tumors probed with AF568-labeled hu-6g8[epitope2] (red); DAPI nuclear-DNA stain (blue), with DIC overlay. Bar = 20 μm. **c** Quantitation of human tumors with > 50% vs < 50% tumor proportion score (TPS) for DEspR+ expression detected by AF568-hu-6g8, *n* = 133 PDAC-patient tumor-cores on tumor array. **d** Quantitation of DEspR expression of invading PDAC TCs in primary tumors with invasive tumor cells in stroma; *n* = 79 PDAC-patient tumor-cores

Analysis of hu-6g8 immunofluorescence in 133-patient PDAC-tumor arrays detected no DEspR expression in normal pancreas (Fig. 6a-b), in contrast to DEspR+ expression in TCs and microvessels in all PDAC stages (I-IV), as well as in invasive TCs in tumor-stroma (Fig. 6a-b). Isotype-IgG4 immunostaining verified specificity of hu-6g8 immunostaining (Fig. 6a). Additionally, we observed DEspR+ expression in the majority of hepatic, omental, and peritoneal metastatic TCs (Fig. 6b). Quantitation by blinded scoring revealed that 82% of PDAC tumor cores with > 50% of tumor proportion scores are DEspR+ (Fig. 6c), and that 90% of 79 tumors with tumor cells invading the stroma are DEspR+ (Fig. 6d).

## Discussion

### DEspR as a nodal therapeutic target in PPC

Cumulative data demonstrate DEspR as a nodal therapeutic target across the PDAC CSC-to-TC spectrum. DEspR is co-expressed in CD133 + ALDH1+ CSCs, and in αSMA+ cscTCs. Blocking anti-DEspR murine precursor and humanized antibodies bind cell-surface DEspR, internalize, and co-translocate with gal1/gal3 nuclear shuttling proteins to the nucleus, subsequently inducing apoptosis in vitro and in vivo. In the PPC-only nude rat model, DEspR-inhibition decreases CSC transperitoneal seeding and subsequent tumor progression and re-dissemination, associated PPC-comorbidities, thereby increasing mOS in both male and female xenografted nude rats.

Improved mOS outcomes from DEspR-inhibition presented here support two key emerging therapeutic paradigms in cancer: a) that reduction of feedforward dissemination-progression requires concurrent inhibition of both ends of the CSC/TC-plasticity spectrum and efficacy in the presence of CSC/TC-heterogeneity, i.e., with DEspR+/− CSCs and TCs [9, 10], and b) that stopping feedforward dissemination-progression in the permissive peritoneal space requires net reduction of pro-survival Mcl1 [31]. We clarify that decreased Mcl-1 protein in ADAR1 KO/DEspR- Panc1 and MiaPaCa2-TCs likely results from DEspR protein-loss, rather than from ADAR1 knockout, since ADARl deficiency increased Mcl-1 in thymocytes [32] whereas DEspR-inhibition decreased Mcl-1 RNA in CSCs [11].

The effects of DEspR-inhibition on suppressing TC- and CSC αSMA expression, and Col1A1 expression/secretion suggest a novel therapeutic paradigm likely contributing to increased mOS in PPC rats via decreasing αSMA-mediated increased invasiveness [33] and collagen-mediated therapy resistance, immune evasion, and invasiveness [34]. These effects are clinically relevant since both αSMA [35] and increased collagen-I [36] are negative prognostic indicators in PDAC patients. The translational potential of DEspR-inhibition is further highlighted by receptor expression in tested human PDAC tumor arrays observed across PDAC stages I-IV and in both primary and metastatic PDAC-patient tumor tissue sections.

### Insights into CSCs in PPC

Specific to the CSC-paradigm in cancer metastasis, the development of PPC after intraperitoneal injection of Panc1-CSCs indicates CSC self-sufficiency in tumor-seeding, and in orchestrating the feed-forward dissemination-progression to pancreatic peritoneal carcinomatosis without requiring prior priming of metastatic beds observed in hematogenous metastases [37]. Specific to CSC-heterogeneity and CSC-subset hierarchy, inhibition of DEspR+ CSCs/TCs, in the presence of DEspR[−] CSCs/TCs, support nodal roles of DEspR+ CSCs/TCs in PPC tumorigenicity and transperitoneal dissemination-progression. Furthermore, conversion of DEspR[−] to DEspR+ CSCs in low adherence stress-culture conditions supports DEspR-roles in anoikis resistance. This also confirms molecular interconversion reported between CSCs subsets [9]. The demonstration of CSC-expression and secretion of Col1A1 independent of fibroblasts or pancreatic stellate cells, and DEspR+ CSC-derived tumor microvessels in vivo [11], suggest CSC self-sufficiency in orchestrating tumorigenesis and progression in the peritoneal space. These data delineate DEspR+ CSC-subset as a nodal driver in transperitoneal dissemination-progression.

### Resolution of DNA-database discordance

In addition to validating the efficacy of DEspR as a therapeutic target in PPC, we show that ADAR1-dependent DEspR expression, via CRISPR/cas9-knockout studies. This reconciles the stop-codon detected in NCBI-DNA databases to cumulative data showing DEspR protein functionality and detection of tryptophan-codon#14-TGG by amplification-refractory mutation system (ARMS)-PCR and in placenta RNA seq-database [18]. These molecular data are further supported by detection of epitope-2, which spans the questioned tryptophan-#14 at epitope-midpoint, by mAbs 6g8 and hu-6g8. We confirm protein function by detection of gal1 and gal3 colocalization, which were previously detected on glycosylated DEspR pull-down experiments [18]. Altogether, data confirm that the DEspR protein is a functional protein upon ADAR1 RNA-editing. Importantly, concordance of pro-cancer roles of both DEspR presented here and prior [11, 18, 38] and ADAR1-editase, and ADAR1-dependency of PDAC cell lines [39] strengthen observations in this study.

## Conclusion

In summary, data showing that anti-DEspR antibody treatment can decrease tumor-seeding and feed-forward dissemination-progression, resulting in significantly increased mOS in both male and female rats with xenografted PPC, collectively suggest a novel therapeutic paradigm for PPC. Coupled with a mode-of-action stabilized by internalization and translocation of hu-6g8/DEspR to the nucleus, data from current (Figs. 1, 2, 3, 4, 5 and 6) and past work [11, 18, 39], collectively support DEspR-inhibition as a translatable therapeutic paradigm. Altogether, data corroborate DEspR protein expression, demonstrate DEspR as a therapeutic target for PPC, and show that the humanized anti-DEspR hu-6g8 IgG4^S228P^ meets the emerging therapeutic paradigm that inhibition of both CSCs and TCs is required to overcome therapeutic resistance arising from CSC-TC plasticity and heterogeneity [9, 10]. These data differentiate hu-6g8 as a potential therapeutic from current standard-of-care therapies for PPC. With patients with PPC having the worst mOS, preclinical efficacy in a PPC nude rat model provides basis to evaluate hu-6g8 in other peritoneal metastatic cancers.

### Limitations of the work

We acknowledge limitations to our studies. We cannot comment on putative differences of DEspR-inhibition between KRAS-mutant vs. wild-type PDAC tumor cells, however we note that > 80–90% of PDAC patients have KRAS mutations which are associated with worse prognosis. Similarly, we cannot comment on preclinical efficacy of DEspR-inhibition in PDX or in immune-competent PPC/PDAC models, however, insights gained in current survival studies using the PPC model will guide future preclinical efficacy studies in different PDAC and metastatic-PDAC models. Furthermore, while we have elucidated part of the downstream targets of DEspR that are decreased upon DEspR-inhibition such as pro-survival protein Mcl-1 in CSCs and TCs, as well as αSMA and Col1A1 in cscTCs and CSCs (presented here), and multipotential CSC-vasculogenesis [11], we recognize that further work remains to be done to dissect each paradigm. Nevertheless, the work presented here comprehensively support DEspR as an important and clinically relevant therapeutic target in PPC, with DEspR-inhibition providing a promising therapeutic approach for patients with pancreatic peritoneal carcinomatosis.

## Supplementary Information

**Additional file 1.** Supplementary Materials and Methods.

**Additional file 2: Fig. S1.** Structural, immunofluorescence, and protein evidence consistent with ADAR1 RNA-editing of DEspR.

**Additional file 3: Fig. S2.** Schematic of ADAR1 knockout (KO) and tumorsphere formation experiment.

**Additional file 4: Table S1.** ADAR1-knockout effects on DEspR+ expression in PDAC tumor cell lines: Panc1 and MiaPaCa2.

**Additional file 5: Table S2.** Comparison of 6g8 and hu-6g8 functionality using different parameters.

**Additional file 6: Fig. S3.** DEspR-hu-6g8 colocalization with galectins-1/3 of Panc1 and MiaPaCa2 TCs.

**Additional file 7: Table S3.** Quantitation of DEspR/gal1 and DEspR/gal3 colocalization in Panc1 and MiaPaCa2 TCs.

**Additional file 8: Table S4.** Study of DEspR inhibition effects on overall survival in Panc1-CDX peritoneal metastasis model.

**Additional file 9: Fig. S4.** Anti-DEspR mAb hu-6g8 demonstrates tumor cell- and tumor-specific target engagement and bioeffects.

## Data Availability

All data analyzed during this study are included in this published article and its supplementary information files. Anti-DEspR antibodies are available to academic researchers with material transfer agreements in compliance with Boston University (BU) policy; and available to industry partners in compliance with BU or option-licensee NControl Therapeutics, Inc.

## Abbreviations

PPC: Pancreatic peritoneal carcinomatosis
mOS: Median overall-survival
CSCs: Cancer stem cells
TC: Tumor cell
PDAC: Pancreatic ductal adenocarcinoma
DEspR: Dual endothelin-1/signal peptide^VEGF^ receptor
cscTCs: CSC-differentiated TCs
IRS: Internalization recognition signal
EMT: Epithelial mesenchymal transformation
Col1A1: Collagen1
ECM: Extracellular matrix
CDX: CSC derived xenograft
IF: Immunofluorescence
dsRNA: Double-strand-RNA
CDRs: Complementarity determining regions
Gal1: Galectin-1
Gal3: Galectin-3
OS: Overall survival (OS)
ip: Intraperitoneal/ly
IHC: Immunohistochemistry
NLR: Neutrophil-lymphocyte ratios
ARMS: Amplification-refractory mutation system
LASC: Lab Animal Science
DMEM: Dulbecco’s Modified Eagle Medium
FBS: Fetal Bovine Serum
KO: ADAR1-knockout
PBS: Phosphate Buffered Saline
AF: Alexa Fluor
HBSS: Hank’s Buffered Salt Solution
ALDH1: Aldehyde dehydrogenase A1
BSA: Bovine serum albumin
HUVECs: Human umbilical vein endothelial cells
mAbs: Monoclonal antibodies
p5: Passage-5
iv: Intravenously
