# Supplementary material for: Humanized anti-DEspR IgG4S228P antibody increases overall survival in a pancreatic cancer stem cell-xenograft peritoneal carcinomatosis ratnu/nu model
Source: BMC Cancer. 2021 Apr 14;21:407. doi: 10.1186/s12885-021-08107-w (PMC8048286; doi:10.1186/s12885-021-08107-w)
Supplement: Supplementary file 1 — Additional file 1. Supplementary Materials and Methods. [file 12885_2021_8107_MOESM1_ESM.pdf]

Additional File 1: Supplementary Materials and Methods

Cell Culture

Panc1 and MiaPaCa2 cells lines were grown in Dulbecco's Modified Eagle Medium (DMEM) (Thermo Scientific 12430-054) supplemented with 10% Fetal Bovine Serum (FBS) (Thermo Scientific 10438026) and 1% Penicillin-Streptomycin (Thermo Scientific 15140122) in a humidified 5% CO2 incubator at 37°C. Cells were harvested at 60% confluence and experiments were performed from cell passage 3-8. CSCs were grown in tumorsphere media: Mammocult media (Stem Cell Technologies 05620), supplemented with 0.0004% Heparin Solution (Stem Cell Technologies, 7980), 0.5% MethoCult (Stem Cell Technologies, 04100), and 1% Penicillin-Streptomycin (Thermo Scientific, 15140122). All CSCs were passaged in non-adherent conditions and experiments performed from passage 3-5. All cscTCs were transformed from passage 4-5 CSCs, with differentiation confirmed by morphological changes observed through microscopy.

Flow Cytometry

For TC experiments, cells were harvested at 60% confluence using pre-warmed 1mM EDTA in PBS with gentle agitation, while CSCs were collected from suspension media by sequential centrifugation, washing, and agitation. Cells were re-suspended in 2% FBS in HBSS and washed thrice to remove media and debris prior to labeling. For measuring cytoplasmic/ nuclear proteins, cells were first fixed with 2% paraformaldehyde for 15 minutes, then washed with ice cold PBS twice followed by permeabilization with 0.1% Triton X-100, and then blocked with 2% FBS in HBSS for 1 hour prior to labeling. Labeling occurred at 4°C in 2% FBS in HBSS for 30 minutes under gentle agitation. Following labeling, cells were filtered using Falcon polystyrene tubes (Thermo Scientific, 0877123), then placed on ice and run immediately

Flow cytometry was performed on an LSRII Flow Cytometer (BD Bioscience) using identical photomultiplier tube voltage settings. Fluorescence minus one and isotypes controls were performed with

collections with appropriate gating. Absolute cell counts were performed with appropriate size-gating. FCS files were analyzed in FlowJo software (Tree Star), with samples gated for singlets by FSC-H, FSC-A, SSC-H versus SSC-A discrimination.

Microscopy

For 7c5-internalization experiments, Panc1 cells were seeded onto Lab-Tek II Chamber Slides at $2.5x10^4$ cells in 1 ml of cell culture media. For all other microscopy experiments, cells were seeded onto MatTek slides at $3x10^4$ cells in 2 ml of cell culture media. For binding studies, media was removed and then cells were washed thrice with ice-cold PBS, and binding with either 7c5-AF568 or hu-6g8-AF568 was performed at 4 ^{o}C under gentle agitation for 20 minutes. Binding without internalization was confirmed by imaging of cells that were washed, then fixed with 2% paraformaldehyde for 20 minutes at 4 ^{o}C. For internalization, cells were washed twice with ice cold PBS, then 1 or 2 ml (depending on slide size) of warmed cell culture media was added. Cells were incubated until appropriate time point, at which point the media was removed, cells were briefly washed with ice cold PBS, and then fixed with 2% paraformaldehyde. For fixed cell microscopy, all confocal microscopy was performed using a TCS SP5 Leica microscope. To achieve adequate signal resolution, a pinhole aperture of 1AU was used. Images were obtained with 0.7 µm Z-stacks.

For live cell imaging, microscopy was performed on an LSM 710-Live Duo Scan confocal microscope (Carl Zeiss) with humidified (37°C, 5% CO_2) Pecon stage-top incubation system. Binding without internalization was first confirmed in cells incubated with ice-cold PBS before treatment with pre-warmed Live Cell Imaging Solution. A 1 AU pinhole aperture was used to obtain adequate signal resolution, with a 63X 1.4 NA plan-apochromat oil immersion objective, with internalization of cells assessed using 0.7µm Z-stacks. Internalization was assessed at 10-min time points. For epifluorescence microscopy, images were taken with identical exposure and capture setting for cell plates and normal controls using ideal settings determined for positive target-specific fluorescence.

For all experiments, appropriate fluorophore labeled isotype controls were used for each fluorescent probe to ensure accurate signal collection. Optimization of fluorescence capture was performed with fluorescence minus one condition to minimize noise, and fluorophores were selected to minimize overlap. For fixed cell microscopy, the following lasers were used for excitation: 405nm (NucBlue), 488nm (AF488), 543nm (AF 546 and AF568), and 633 nm (AF647), and the following windows were set to capture emission: 430-470nm (NucBlue), 505-550nm (AF488), 580-620 (AF546), 615-700nm (AF568), and 700-750 (AF647) to prevent overlap. For live cell microscopy, the following lasers were used for excitation: 405 nm (NucBlue) and 543 nm (AF568), and the following windows were set to capture emission: 430-475 nm (NucBlue) and 600-700 nm (AF568) to prevent overlap.

For all images, fluorescence signal was corrected and background was subtracted using the rolling-ball algorithm for background correction. Image files were analyzed in ImageJ software.

For Live cell imaging, all experiments were performed on Fluorescence signal was corrected and background was subtracted using the rolling-ball algorithm for background correction. Images were analyzed with ImageJ software.

Quantification of Galectin-DEspR Colocalization.

First, fluorescence signal was corrected and background was subtracted using rolling-ball algorithm. Next, colocalization was tested using van Steensel's cross correlation function [1], which provides a qualitative assessment of colocalization. Pearson coefficients are generated as the signal from one channel is moved along the x-axis of the other channel, then plotted as a function of the x-axis. Colocalization is confirmed if the overlay of both images at the origin represents the maximal Pearson coefficient. Once colocalization was confirmed, quantitative analysis was performed by calculating the overlap coefficient, as proposed by Manders and colleagues [2]. First, a correlation coefficient, *r*, is calculated, which is modified from the Pearson coefficient, given in the below formula:

$$r = \frac{\sum_i A_i \cdot B_i}{\sqrt{\sum_i A_i^2 \cdot \sum_i B_i^2}}$$

where A is the gray value from one fluorescent channel and B is the gray value of another fluorescent channel in a specific analyzed space, or voxel. This correlations coefficient, r, however, does not provide information on the relative strength of association of A to B or B to A. Therefore, we calculated individual colocalization coefficients, the Mander's colocalization coefficients, which explain this association, κ [2]. For signal A, this is defined as:

$$k_{A \to B} = \frac{\sum_i A_{i,coloc}}{\sqrt{\sum_i A_i}}$$

and for signal B, this is defined as:

$$k_{B \to A} = \frac{\sum_i B_{i,coloc}}{\sqrt{\sum_i B_i}}$$

Where $A_{i,coloc} = A_i$ when $B_i > 0$; and $B_{i,coloc} = B_i$ when $A_i > 0$; and $A_{i,coloc} = 0$ if $B_i = 0$; and $B_{i,coloc} = 0$ when $A_i = 0$. For our analysis, we focused on the first association, that being $k_{DEspR\text{-}gal}$ as the association of DEspR to the galectin signal, in order to measure how much of the galectin signal was colocalized with DEspR. Cells were considered to have DEspR colocalized with a particular galectin when $k_{DEspR\text{-}gal}$ was greater than 0.5, suggesting a stronger, non-random association. Cells that had internalized hu-6g8-DEspR complex but no colocalization ($k < 0.5$) with either gal1 or gal3 were noted, suggesting gal1/3-independent cytoplasmic shuttling.

The same technique was employed for analyzing nuclear colocalization, by comparing the Manders colocalization coefficient, κ, of the DEspR-hu-6g8 complex with either galectin.

CRISPR/Cas9 ADAR1 KO

For ADAR1 KO, Panc1 and MiaPaCa2 TCs were seeded at 3 x 10^5 cells in 2 ml cell media per well using a 6-well tissue culture plates (Fisher Scientific 07-200-83), and allowed to grow to 50% confluence. Media was then changed to antibiotic free, serum-free growth media 24 hours prior to transfection. A

solution of 1.5 μg CRISPR/Cas9 KO (h) plasmid (SantaCruz sc-401611) and 1.5 μg ADAR1 Homology-Directed Repair (HDR) plasmid (h) (SantaCruz sc-401611) was prepared in plasmid transfection media (SantaCruz sc-108062), and mixed with a solution of 10 μl UltraCruz Transfection Reagent (SantaCruz sc-395739) in plasmid transfection media. This solution was added dropwise to each well; after 6 hours, media was replaced. After 24 hours, complete growth media (with serum and Penicillin/Streptomycin antibiotics) was added. For some cells, the plate was trypsinized and re-plated to separate the cells and improve CRISPR/Cas9 efficiency. Media was changed every 24 hours to remove dead cells; once transfected cells reached 80% confluence, complete growth media with 5 μg/ml puromycin was added to the plate. Puromycin-containing media was changed every 24 hours for passage (p)2-3, then every 48 hours for all subsequent passages, based on the growth of the tumor cells, up to p5. Passage 1-2 took 7 days, from passage 3-5, each passage was 5 days. Transfection efficacy was assessed by monitoring GFP expression in vector positive cells, using a Keyence BZ-9000 fluorescence microscope. Flow cytometry was performed to assess vector cell positive (via GFP expression) as well as, ADAR1 and DEspR cell expression at each passage. This same protocol was used for the murine ADAR1 HDR plasmid (m) as mock-transfection and puromycin selection control. Plasmid and transfection reagent concentrations were optimized for Panc1 and MiaPaCa2 respectively. Optimal conditions were identical for both Panc1 and MiaPaCa2. A graphical representation of the experimental design is provided in Supplemental Figure S-2A, and Figure S-2B.

Animal Models

All studies used Rowett nude$^{nu/nu}$ rats obtained from Charles River Labs, ordered at minimum 1 week prior to proposed studies to allow accommodation to animal facilities. An established IACUC protocol [AN15160] was in place for all animal studies with agreed upon endpoints. Rats were assigned to study groups based on pre-study design schemes, monitored twice daily for health concerns, with body weights obtained weekly. Observing Lab Animal Science Center (LASC) monitors at BU-LASC were blinded to treatment groups, and decision to euthanize was based on LASC observation with strict criteria to

euthanize if rats lost >10% of maximum body weight or had <10% total body weight loss but significant comorbidities where survival would be <24hrs. At end-stage, rats were assessed for comorbidities and euthanized with photo-documentation of ascites, peritoneal tumors and abdominal organ states, and tumor and organ collection for further analysis. For all studies nude rats were age- and weight-matched, equivalently assigned to study groups using pre-study algorithm to equivalently distribute body weight. Procedures were performed under isoflurane anesthesia, with 3% for induction and 1% for maintenance dissolved in medical air, to minimize, if not eliminate, animal stress. For heterotopic subcutaneous Panc1 PPC model, antibody doses were selected based on binding studies to CSCs. Observed maximal DEspR binding was seen at 10 μg/ml for 100,000 cells, therefore 200 μg/ml was selected for 20x cell concentration. Rats were monitored for tumor volumes. For all pre-treatment studies, no further interventions were performed and no blood was collected. For both female treatment studies, blood was obtained at 28, 35, and 42 days post-injection; no blood was obtained from male study. MiaPaCa2 CSCs also produced PPC tumors in nude-rats but the xenografting penetration was <100% despite using identical conditions to Panc1-CSC derived PPC nude rat model, hence survival studies were aborted.

Pharmacokinetic Analysis of hu-6g8

Serum samples were analyzed using Western blot and quantified with Image J relative to known standards to determine serum analyte content. Data was plotted relative to time and analyzed in a series of one and two-compartment models using PKSolver software [3], fit to a single-bolus injection. Parameters fit to a two-compartment model, depicting rapid internalization and distribution.

Tissue Immunofluorescence

Paraffin was removed by heating at either 60 °C or 65 °C for 30 minutes to 1 hour, depending on section thickness, followed by and submersion in xylene, then gradually rehydrated in alcohol dilutions. Antigen retrieval was performed with 0.1 M Na-phosphate buffer (pH = 6.8) at 95 °C for 20 minutes. Blocking was performed with 5% BSA in PBS solution for 2 hours. Slides were then treated with Image-iT Fx

Signal Enhancer (ThermoFisher, I36933) per manufacturer instructions prior to staining. Following labeling done at 4 °C overnight, sections were washed then mounted with ProLong Diamond Antifade Mountant with DAPI (ThermoFisher, P36962) per manufacturer instructions.

Equivalence Determination of Gemcitabine Dosing in Rats and Mice

Since rats have 7.6-fold longer gemcitabine half-life compared to mice [4], the rat dose of 26 mg/kg/dose is equivalent to a mouse dose of 198 mg/kg/dose. For the rat dose of 100 mg/kg/dose that results in severe myelosuppression and lethality after a 2nd dose in the PPC rats, the equivalent mouse dose is 760 mg/kg/dose, which is close to the maximum tolerated dose of 700 mg/kg/dose determined in mice for gemcitabine [5].

Reproducibility

To ascertain reproducibility of *in vitro* study results, we used independent experimental systems and parameters. *In vitro* experiments were done with independent biological replicates (different days, as well as different experimenters) in addition to technical replicates to demonstrate methodological rigor. We used different levels of biological replicates: a) two PDAC cell lines, and b) different cell types: CSCs, non-CSC-TCs, and csc-TCs, and mixed pool CSCs, tumor cells, ie, both DEspR+ and DEspR(-) cells. This better represents the heterogeneity in human tumors, and validates the experimental unit as each cell in a mixed pool for flow cytometry, functional assays *in vitro*, aside from independent experimental set-up in different wells, different days and experimenters. We also used different informative, functional endpoints (such as, apoptosis over different timepoints, mAb-receptor internalization, nuclear translocation, co-localization with nuclear shuttling proteins, function shutdown of αSMA and Col1A1 expression), thus ascertaining reproducibility of DEspR-inhibition efficacy on attenuating PDAC cancer cells. We used one parameter per experiment, so every experimental unit was an observational unit, fulfilling true replication. We used two PDAC cell lines selected with different KRAS mutations, Panc1 and MiaPaCa2, from authenticated ATCC stocks.

To ascertain reproducibility, all *in vivo* xenograft model experiments in this report were assigned to corresponding study groups ascertaining equivalent littermate distribution across study groups, and equivalent average body weights among the different study groups. Twice daily monitoring was performed by Lab Animal Science technicians blinded to the study group assignments, and performed determination of need for euthanasia in blinded manner according to approved IACUC protocol specifications. Controls and test groups were matched as to level of biological endpoints, with distinct study groups per biological parameter measured. Each survival study had its own independent study cohorts, which were performed on different PPC-xenografted rat groups, at different periods, and by different experimenters, thus demonstrating that PPC-modeling is reproducible and robust.

To ascertain reproducibility of efficacy analysis, we measured objective endpoint parameters: a) overall survival, b) tumor burden, and c) target engagement and bioeffects in distinct and independent cohorts with corresponding appropriate controls. These endpoint parameters are robust biological parameters with no potential to be affected by cagemates. We used outbred Rowett nude rats, not inbred nude mice, in PPC-xenograft modeling in order to obtain greater tumor burden to recapitulate comorbidities, and to better represent genetic diversity and more stringently test efficacy of DEspR-inhibition in the presence of genetic diversity as would be seen in humans. We monitored for adverse events in all efficacy studies.

To ascertain reproducibility in PPC-modeling, all PPC xenografted study rats were injected at the same age, with identical number of CSCs isolated at identical passage-number. All study PPC-xenografted rats were palpated to verify presence of peritoneal tumors at start of treatment, and all treatments were given independently via individual intravenous infusion. Intravenous administration of treatment vs control vehicle was done under isoflurane anesthesia to eliminate animal stress, as well as eliminate confounders and variability from environmental stresses. Controls and treated rats were all run contemporaneously to eliminate seasonal environmental confounders.

To ascertain reproducibility of DEspR treatment efficacy, *in vivo* efficacy studies were performed with more than one anti-DEspR antibody using targeting different epitopes on DEspR protein (7c5, 5g12,

6g8), different IgG backbones (murine and human IgG), and in both male and female PPC-xenograft tumor rats.

Institutional Compliance for Animal Model Use for Research

In vivo animal model research studies were designed, performed, analyzed and reported in compliance with institutional guidelines for vertebrate animal research at Boston University [IACUC protocol AN15160] as subcontract to Abtelum Biomdeical, Inc., in accordance with National Institutes of Health-National Cancer Institute SBIR-vertebrate animal research review, and ARRIVE 2.0 guidelines [6]. The Lab Animal Science Center at Boston University is accredited by the American Association for Accreditation of Laboratory Animal Care. The AN15160 IACUC protocol was approved prior to onset of animal model research studies.

SUPPLEMENTARY MATERIALS AND METHODS REFERENCES

1. Van Steensel B, van Binnendijk EP, Hornsby CD, van der Voort HT, Krozowski ZS, de Kloet ER, et al. Partial colocalization of glucocorticoid and mineralocorticoid receptors in discrete compartments in nuclei of rat hippocampus neurons. J Cell Sci. 1996;109:787-92.
2. Manders EM, Stap J, Brakenhoff GJ, van Driel R, Aten JA. Dynamics of three-dimensional replication patterns during the S-phase, analysed by double labelling of DNA and confocal microscopy. J Cell Sci. 1992;103:857-62.
3. Zhang Y, Huo M, Zhou J, Xie S. PKSolver: an add-in program for pharmacokinetic and pharmacodynamic data analysis in Microsoft Excel. Comput Methods Programs Biomed. 2010;99:306-14.
4. Shipley LA, Brown TJ, Cornpropst JD, Hamilton M, Daniels WE, Culp HW. Metabolism and disposition of gemcitabine, and oncolytic deoxycytidine analog, in mice, rats, and dogs. Drug Metab Dispos. 1992;20:849-55.

5. Aston WJ, Hope DE, Nowak AK, Robinson BW, Lake RA, Lesterhuis WJ. A systematic investigation of the maximum tolerated dose of cytotoxic chemotherapy with and without supportive care in mice. BMC Cancer. 2017;17:684.
6. Percie du Sert N, Hurst V, Ahluwalia A, Alam S, Avey MT, Baker M, Browne WJ, Clark A, Cuthill IC, Dirnagl U, Emerson M, Garner P, Holgate ST, Howells DW, Karp NA, Lazic SE, Lidster K, MacCallum CJ, Macleod M, Pearl EJ, Petersen O, Rawle F, Peynolds P, Rooney K, Sena ES, Silberberg SD, Steckler T and Wurbel H (2020). The ARRIVE guidelines 2.0: updated guidelines for reporting animal research. *PLoS Biol.* doi: 10.1371/journal.pbio.3000410
