# Supplementary material for: Humanized anti-DEspR IgG4S228P antibody increases overall survival in a pancreatic cancer stem cell-xenograft peritoneal carcinomatosis ratnu/nu model
Source: BMC Cancer. 2021 Apr 14;21:407. doi: 10.1186/s12885-021-08107-w (PMC8048286; doi:10.1186/s12885-021-08107-w)
Supplement: Supplementary file 2 — Additional file 2: Fig. S1. Structural, immunofluorescence, and protein evidence consistent with ADAR1 RNA-editing of DEspR. [file 12885_2021_8107_MOESM2_ESM.pdf]

**Additional File 2: Fig. S1. Structural, immunofluorescence, and protein evidence consistent with ADAR1 RNA-editing of DEspR.**

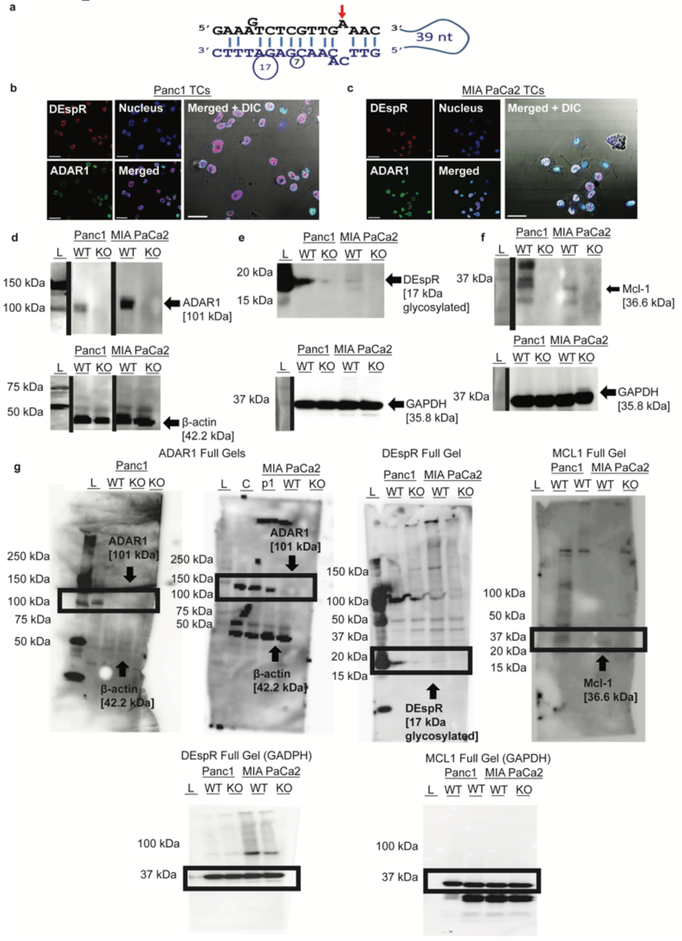

**Figure S1. Structural, immunofluorescence, and protein evidence consistent with ADAR1 RNA-editing of DEspR. (a)** Predicted 15-bp minimum double-strand RNA hairpin loop structure required for ADAR1 site-specific editing of DEspR mRNA, centered on the A-I(G) RNA edited site for Tryp-14 codon. **(b)** Representative high-power field (HPF) confocal micrographs showing ADAR1 and DEspR expression in Panc1 TCs (n=61 cells, 3 HPFs; Pearson's coefficient r of DEspR-ADAR1 colocalization correlation (r = 0.844). **(c)** Representative HPF confocal micrograph showing ADAR1 and DEspR expression in MiaPaCa2 TCs (n = 38 cells, 3 HPFs; Pearson's r = 0.748). Legend: DEspR (red), ADAR1 (green), and DAPI+nucleus (blue); merged (Right) shows ADAR1 and DEspR colocalization (yellow), DEspR/DAPI+ colocalization (magenta), and colocalization of all three signals (white). DIC: differential interference contrast. Scale bar: 40um. **(d-f)** Western blot analyses comparing Panc1 and MiaPaCa2 ADAR1 WT *vs* KO at passage 5 of: ADAR1 (Panc1 and MiaPaCa2 run on separate gels- denoted by line; ladder (L) from MiaPaCa2 gel for better contrast), **(d),** DEspR **(e),** and Mcl-1 (line separating ladder, which is at higher contrast to show marker lines) **(f)**. Control proteins selected to eliminate size-overlaps: β-actin for ADAR1 (line denotes separate gels as above), and GAPDH for Mcl1 and DEspR (ladder used at different contrast given intensity of GAPDH signal). Controls were probed on the same protein-blot after stripping off the test-protein signal. **(g)** Full blots of ADAR1 of Panc1 (showing WT vs KO), MiaPaCa2 (showing control murine KO (c), passage-1 of KO (p1), WT vs. KO), DEspR expression, and Mcl1 expression. GAPDH controls for DEspR and Mcl shown below. β-actin controls for Panc1 and MiaPaCa2 shown from previous control after attempted stripping of gel.

ADAR1-KO TCs showed decreased expression of all test-proteins relative to wild type. **ADAR1.** Panc1: 8.5% ± 4.1% of WT; MiaPaCa2: 9.0% ± 1.3% of WT. **DEspR** (hu-6g8 probe). Panc1: 9.4% ± 1.3% of WT; MiaPaCa2: 17.5% ± 6.4% of WT. **Mcl-1.** Panc1: 10.4% ± 1.8% of WT; MiaPaCa2: 32.9% ± 5.6% of WT.
