# Supplementary material for: Humanized anti-DEspR IgG4S228P antibody increases overall survival in a pancreatic cancer stem cell-xenograft peritoneal carcinomatosis ratnu/nu model
Source: BMC Cancer. 2021 Apr 14;21:407. doi: 10.1186/s12885-021-08107-w (PMC8048286; doi:10.1186/s12885-021-08107-w)
Supplement: Supplementary file 3 — Additional file 3: Fig. S2. Schematic of ADAR1 knockout (KO) and tumorsphere formation experiment. [file 12885_2021_8107_MOESM3_ESM.pdf]

Additional File 3: Fig. S2. Schematic of ADAR1 knockout (KO) and tumorsphere formation experiment

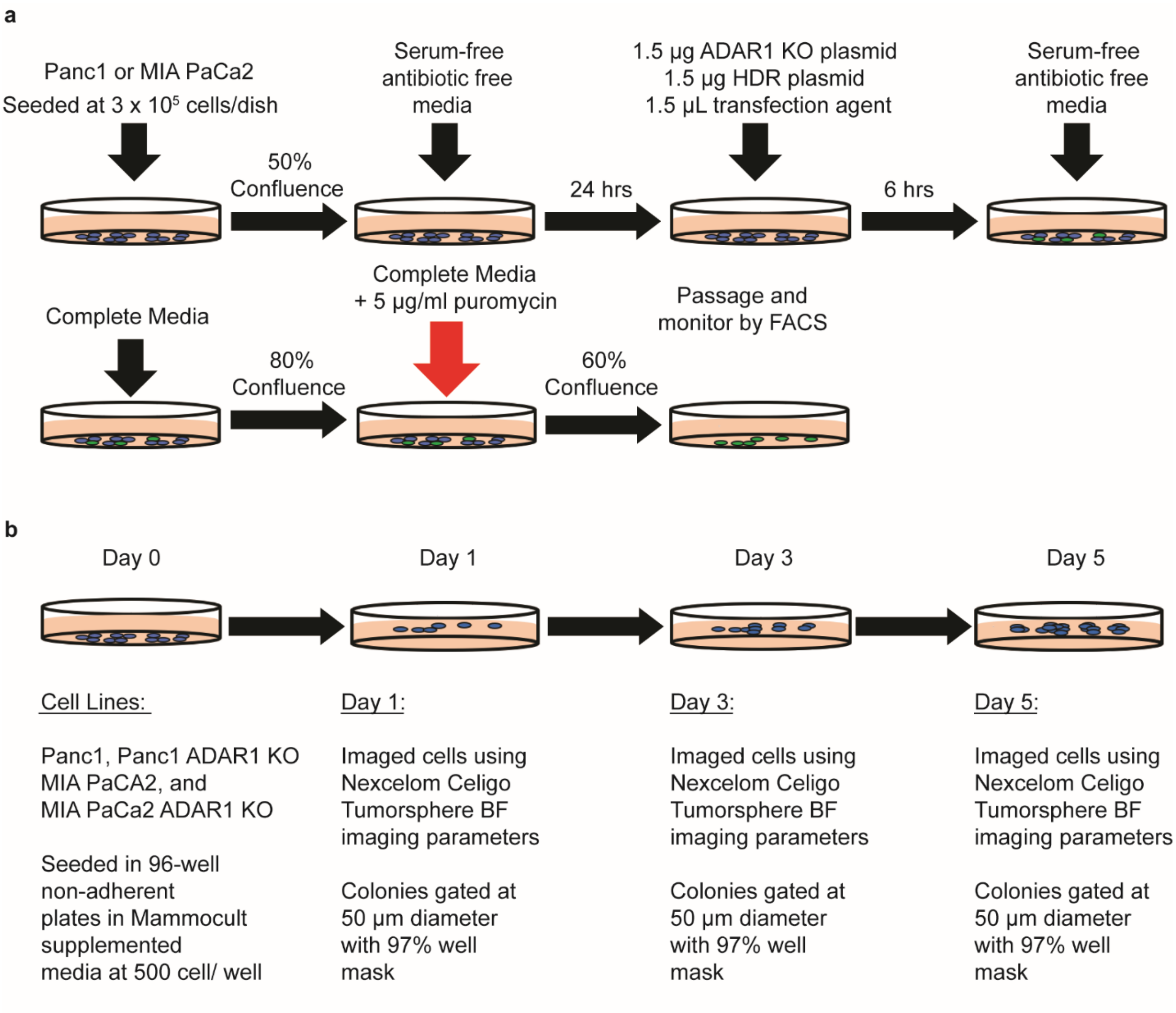

Figure S2. Schematic of ADAR1 knockout (KO) and tumorsphere formation experiment. (a) Graphical representation of CRISPR/Cas9 ADAR1 KO experiment using Santa Cruz high efficiency knockout plasmids and homologous domain repair (HDR) selection plasmid for preparation of transfected ADAR1-KO Panc1 and MiaPaCa2 cells. **(b)** Schematic of tumorsphere-formation assay of Panc1-WT and Panc1-KO, and MiaPaCa2-WT and MiaPaCa1-KO TCs.
