# Supplementary material for: Humanized anti-DEspR IgG4S228P antibody increases overall survival in a pancreatic cancer stem cell-xenograft peritoneal carcinomatosis ratnu/nu model
Source: BMC Cancer. 2021 Apr 14;21:407. doi: 10.1186/s12885-021-08107-w (PMC8048286; doi:10.1186/s12885-021-08107-w)
Supplement: Supplementary file 4 — Additional file 4: Table S1. ADAR1-knockout effects on DEspR+ expression in PDAC tumor cell lines: Panc1 and MiaPaCa2. [file 12885_2021_8107_MOESM4_ESM.pdf]

Additional File 4: Table S1. ADAR1-knockout effects on DEspR+ expression in PDAC tumor cell lines: Panc1 and MiaPaCa2

PDAC cell line	Passage	DEspR+/ADAR1+	DEspR+ Only	ADAR1+ Only
**Panc1**	Baseline	98.4 ± 0.57 %	1.46 ± 0.50 %	0.01 ± 0.00 %
	P3	0.20 ± 0.01 %	98.60 ± 0.14 %	0.01 ± 0.00 %
	P4	0.00 ± 0.00 %	0.03 ± 0.03 %	0.02 ± 0.00 %
**MiaPaCa2**	Baseline	97.1 ± 0.60 %	2.82 ± 0.56 %	0.00 ± 0.00 %
	P3	4.61 ± 0.17 %	94.75 ± 0.21 %	0.00 ± 0.01 %
	P4	0.00 ± 0.00 %	0.031 ± 0.05 %	0.02 ± 0.01 %

The % of cells expressing ADAR1 (ADAR1+) and/or DEspR (DEspR+) detected on flow cytometry of Panc1 and MiaPaCa2 at baseline and after CRISPR/Cas9 ADAR1 knockout at passage-3 (p3) and passage-4 (p4).
