# Supplementary material for: Humanized anti-DEspR IgG4S228P antibody increases overall survival in a pancreatic cancer stem cell-xenograft peritoneal carcinomatosis ratnu/nu model
Source: BMC Cancer. 2021 Apr 14;21:407. doi: 10.1186/s12885-021-08107-w (PMC8048286; doi:10.1186/s12885-021-08107-w)
Supplement: Supplementary file 5 — Additional file 5: Table S2. Comparison of 6g8 and hu-6g8 functionality using different parameters. [file 12885_2021_8107_MOESM5_ESM.pdf]

Additional File 5: Table S2. Comparison of 6g8 and hu-6g8 functionality using different parameters.

	Murine 6g8	hu-6g8
EC_{50}: binding to DEspR on intact Panc1 TCs	5.6 ± 0.25 µg/ml	0.6 ± 0.25 µg/ml
B_{max} for binding to DEspR	35.6 ± 4.0 µg/ml	31.5 ± 2.6 µg/ml
IC_{50}: inhibition of Panc1 CSC survival in anoikis conditions	>> 30.0 µg/ml	1.2 ± 0.12 µg/ml
B_{max} for inhibition of Panc1 CSC survival	< 10 µg/ml	44.1 ± 5.6 µg/ml
IC_{50}: inhibition of angiogenesis HUVEC tube formation	16.6 ± 5.5 µg/ml	0.52 ± 0.05 µg/ml
B_{max} for inhibition of angiogenesis	37.1 ± 21 µg/ml	38.0 ± 5.0 µg/ml

B_{max}, calculated maximum receptor number; CSC, cancer stem cells; DEspR, dual endothelin-1/signal ^{VEGF} peptide receptor; EC_{50}, effective concentration of antibody that gives half-maximal response between the baseline and maximum response; HUVEC, human umbilical vein endothelial cells; $IC_{50,}$ effective concentration of antibody where the response is reduced by half; TCs, tumor cells; µg/ml, microgram per ml.
