# Supplementary material for: Humanized anti-DEspR IgG4S228P antibody increases overall survival in a pancreatic cancer stem cell-xenograft peritoneal carcinomatosis ratnu/nu model
Source: BMC Cancer. 2021 Apr 14;21:407. doi: 10.1186/s12885-021-08107-w (PMC8048286; doi:10.1186/s12885-021-08107-w)
Supplement: Supplementary file 6 — Additional file 6: Fig. S3. DEspR-hu-6g8 colocalization with galectins-1/3 of Panc1 and MiaPaCa2 TCs. [file 12885_2021_8107_MOESM6_ESM.pdf]

Additional File 6: Fig. S3. DEspR-hu-6g8 colocalization with galectins-1/3 of Panc1 and MiaPaCa2 TCs

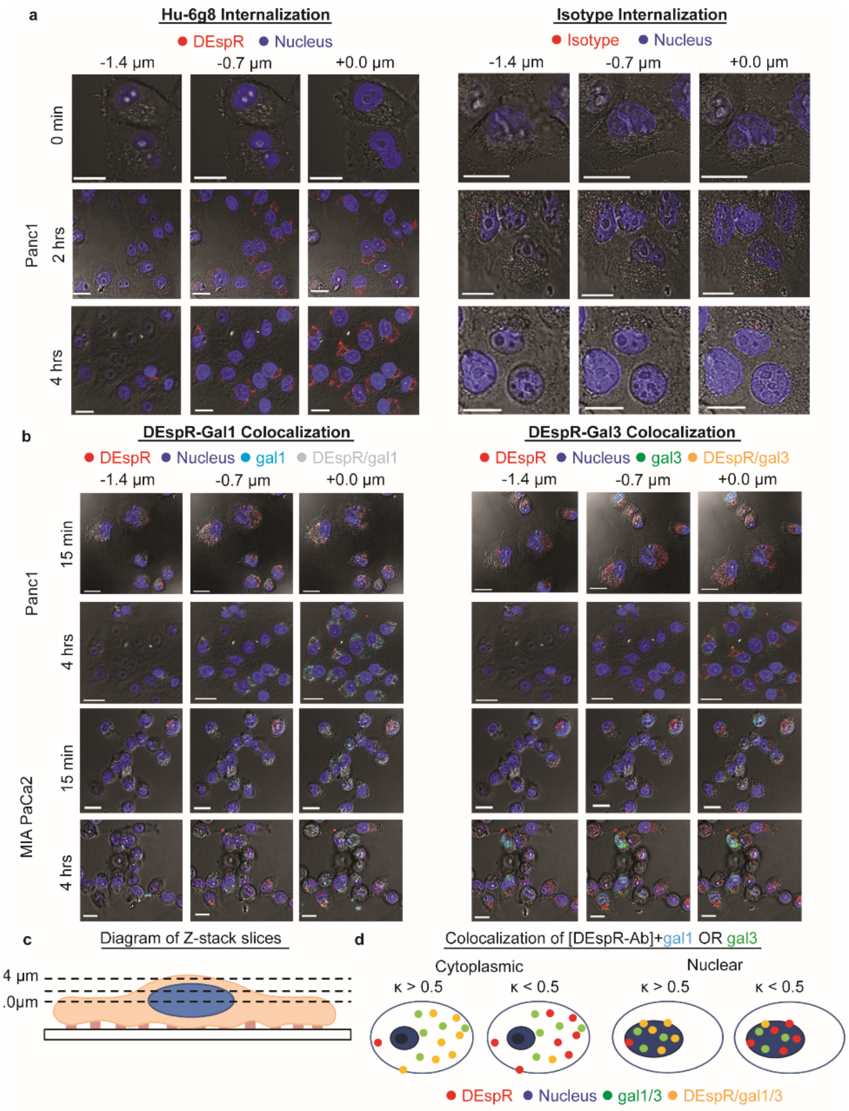

Figure S3. DEspR-hu-6g8 colocalization with galectins-1/3 of Panc1 and MiaPaCa2 TCs. (a) Representative Z-stack confocal images of hu-6g8 internalization [**Left**] compared to isotype internalization [**Right**], at fixed time points. Binding at $4^{o}C$ (t=0 min), shows binding to cell surface only, without internalization, while later time points show nuclear colocalization (Z = +0.0 μm representing mid-nuclear level) in hu-6g8 internalization. **(b)** Multiplex immunofluorescence of [**Left**] Panc1 and [**Right**] MiaPaCa2 TCs treated with hu-6g8 and probed with human-specific AF568-anti-IgG to detect hu-6g8/DEspR ab/receptor complexes, AF546-anti-gal1 and AF488-anti-gal3 to assess colocalization with hu-6g8/DEspR. Representative Z-stacks showing transport into the cell towards the nueleus. Legend: hu-6g8/DEspR complex (red), gal1 (aqua), gal3 (green), gal1/DEspR colocalization (white), gal3/DEspR colocalization (yellow), and DEspR/DAPI+DNA colocalization (magenta). Bar=20μm **(c)** Diagram of confocal microscopy Z-stacks, 0.0 μm at nuclear midpoint-level, -1.4 μm close to surface. **(d)** Diagram of quantitative analysis of colocalization of hu-6g8/DEspR complex with gal1/gal3 in the cytoplasm [**Left**], or in the nucleus [**Right**] using Manders' overlap coefficient: $\kappa>0.5$ indicates colocalization; $\kappa<0.5$ indicated minimal colocalization. Legend: hu-6g8/DEspR complex (red), gal1/3 (green), colocalized gal1/3-[DEspR-Ab] complex (yellow). Quantitative analyses of cytoplasmic and nuclear DEspR/gal1 and DEspR/gal3 colocalization were performed using Manders' coefficients of colocalization, r and κ. (Table S3).
