# Supplementary material for: Humanized anti-DEspR IgG4S228P antibody increases overall survival in a pancreatic cancer stem cell-xenograft peritoneal carcinomatosis ratnu/nu model
Source: BMC Cancer. 2021 Apr 14;21:407. doi: 10.1186/s12885-021-08107-w (PMC8048286; doi:10.1186/s12885-021-08107-w)
Supplement: Supplementary file 7 — Additional file 7: Table S3. Quantitation of DEspR/gal1 and DEspR/gal3 colocalization in Panc1 and MiaPaCa2 TCs. [file 12885_2021_8107_MOESM7_ESM.pdf]

Additional File 7: Table S3. Quantitation of DEspR/gal1 and DEspR/gal3 colocalization in Panc1 and MiaPaCa2 TCs.

		Cytoplasmic colocalization				Nuclear colocalization			
Panc1	*Time (min)*	$r_{DEspR\text{-}gal1}$	$\kappa_{DEspR\text{-}gal1}$	$r_{DEspR\text{-}gal3}$	$\kappa_{DEspR\text{-}gal3}$	$r_{DEspR\text{-}gal1}$	$k_{DEspR\text{-}gal1}$	$r_{DEspR\text{-}gal3}$	$k_{DEspR\text{-}gal3}$
	15	0.507	0.834	0.180	0.223	0.395	0.940	0.200	0.632
	30	0.450	0.982	0.154	0.240	0.312	0.485	0.142	0.441
	120	0.452	1.103	0.220	0.308	0.335	0.716	0.266	0.809
	240	0.429	0.541	0.255	0.246	0.305	0.336	0.312	1.036
MiaPaCa2	15	0.318	0.594	0.188	0.432	0.327	0.545	0.188	0.652
	30	0.445	0.570	0.332	0.461	0.345	0.790	0.280	0.717
	120	0.328	0.760	0.257	0.748	0.221	0.680	0.201	1.060
	240	0.313	0.234	0.257	0.748	0.171	0.050	0.364	0.661

Colocalization is quantified using ***r***, a correlation coefficient of colocalization, which is modified from the Pearson coefficient; and κ, Manders overlap coefficient which measures the relative strength of association - how much of DEspR-hu-6g8 associates with each galectin (gal1/gal3) while also factoring in non-association. Analysis by JaCoP software plug-in on ImageJ; see Supplementary Methods
