# Supplementary material for: Humanized anti-DEspR IgG4S228P antibody increases overall survival in a pancreatic cancer stem cell-xenograft peritoneal carcinomatosis ratnu/nu model
Source: BMC Cancer. 2021 Apr 14;21:407. doi: 10.1186/s12885-021-08107-w (PMC8048286; doi:10.1186/s12885-021-08107-w)
Supplement: Supplementary file 8 — Additional file 8: Table S4. Study of DEspR inhibition effects on overall survival in Panc1-CDX peritoneal metastasis model. [file 12885_2021_8107_MOESM8_ESM.pdf]

Additional File 8: Table S4. Study of DEspR inhibition effects on overall survival in Panc1-CDX peritoneal metastasis model.

Study	Comparators	Dose Regimen	*p*	N	MS (days)
Multi-dose (Female)	***mu-6g8***	1 mg/kg ip, 2/wk x 4 wks	0.0002	8	> 120
	mu-7c5	1 mg/kg ip, 2/wk x 4 wks	0.002	7	> 120
	Gemcitabine	28 mg/kg ip, 1/wk x 4 wks	n.s. [0.126]	7	66.0
	Saline	1 ml ip, 1/wk x 4 wks	Reference	15	43.0
Single-dose (Female)	***hu-6g8***	3 mg/kg iv x 1 dose	0.001	8	92.0
	hu-6g8	15 mg/kg iv x 1 dose	0.0007	7	189.0
	Gemcitabine	100 mg/kg iv x 1 dose	0.0018	7	135.0
	Saline	1 ml ip x 1 dose	Reference	12	62.0
Single-dose (Male)	***hu-6g8***	15 mg/kg iv x dose	0.02	6	98.0
	Saline	1 ml ip x 1 dose	Reference	6	72.0

MODEL: Panc1-CDX PPC model in RNU/RNU 'nude' rats
TREATMENT ONSET: ~ 3 weeks after CSC injection, palpable omental metastatic tumors
LEGEND: CDX, CSC-derived xenograft tumor model.
Administration: iv, intravenous route via tail vein; ip, intraperitoneal route of administration; dose in mg per kg, kilogram body weight.
Columns: p values relative to saline; MS- median survival in days, N- sample size per group.
Mantel-Cox log rank test followed by Holm-Sidak multiple comparisons testing when > 2 study groups; PPC, CDX-pancreatic peritoneal carcinomatosis model in nude rats; wk(s), weeks
