# Supplementary material for: Humanized anti-DEspR IgG4S228P antibody increases overall survival in a pancreatic cancer stem cell-xenograft peritoneal carcinomatosis ratnu/nu model
Source: BMC Cancer. 2021 Apr 14;21:407. doi: 10.1186/s12885-021-08107-w (PMC8048286; doi:10.1186/s12885-021-08107-w)
Supplement: Supplementary file 9 — Additional file 9: Fig. S4. Anti-DEspR mAb hu-6g8 demonstrates tumor cell- and tumor-specific target engagement and bioeffects. [file 12885_2021_8107_MOESM9_ESM.pdf]

Additional File 9: Fig. S4. Anti-DEspR mAb hu-6g8 demonstrates tumor cell- and tumor-specific target engagement and bioeffects.

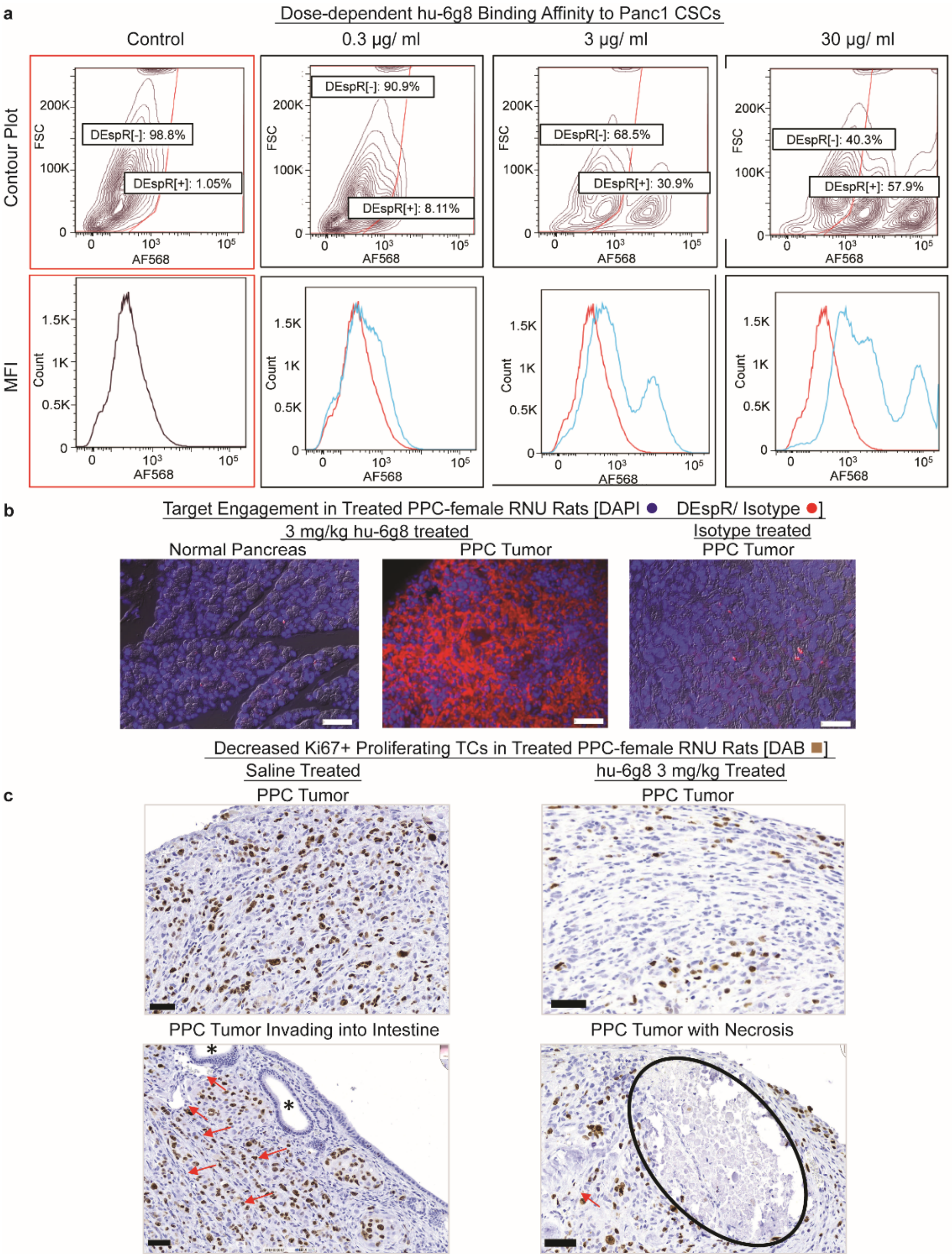

Figure S4. Anti-DEspR mAb hu-6g8 demonstrates tumor specificity with minimal off-target effect. (a). Tumor cell target engagement: Flow-cytometry analysis of hu-6g8 binding to DEspR on live Panc1 CSCs showing concentration-dependent binding affinity. **(b).** Tumor-specific target engagement *in vivo*: 3 mg/kg hu-6g8 treatment does not bind to normal pancreas [**Left**]; in contrast to tumor-target engagement in PPC tumor [**Middle**], and in contrast to no tumor target-engagement of control isotype IgG4 [**Right**]. Bar=20μm. **(c)** Comparison of cell proliferation in **[Left]** saline-treated and **[Right]** hu-6g8 treated PPC rats, measured by DAB+ (brown) IHC-staining for Ki67. Hu-6g8 treated tumors showed areas with decreased Ki67+proliferation, decreased tumor microvessels (red ➔), and areas of cell loss (black circle) compared to isotype controls. Bar = 50μm.
